# Supplementary material for: Clinical utility of peptide microarrays in the serodiagnosis of neglected tropical diseases in sub-Saharan Africa: protocol for a diagnostic test accuracy systematic review
Source: BMJ Open. 2021 Jul 30;11(7):e042279. doi: 10.1136/bmjopen-2020-042279 (PMC8327806; doi:10.1136/bmjopen-2020-042279)
Supplement: Author's manuscript [file bmjopen-2020-042279.draft_revisions.pdf]

BMJ Open is committed to open peer review. As part of this commitment we make the peer review history of every article we publish publicly available.

When an article is published we post the peer reviewers’ comments and the authors’ responses online. We also post the versions of the paper that were used during peer review. These are the versions that the peer review comments apply to.

The versions of the paper that follow are the versions that were submitted during the peer review process. They are not the versions of record or the final published versions. They should not be cited or distributed as the published version of this manuscript.

BMJ Open is an open access journal and the full, final, typeset and author-corrected version of record of the manuscript is available on our site with no access controls, subscription charges or pay-per-view fees (http://bmjopen.bmj.com).

If you have any questions on BMJ Open’s open peer review process please email info.bmjopen@bmj.com

BMJ Open

Clinical utility of peptide microarrays in the serodiagnosis and surveillance of neglected tropical diseases in sub-Saharan Africa: Protocol for a diagnostic test accuracy systematic

Journal:	*BMJ Open*
Manuscript ID	bmjopen-2020-042279
Article Type:	Protocol
Date Submitted by the Author:	20-Jul-2020

Complete List of Authors:	Vengesai, Arthur; University of Zimbabwe Faculty of Science, Biochemistry; University of KwaZulu-Natal College of Health Sciences Durban, ZA, Optics and Imaging Centre<br>Naicker, Thajasvarie; University of KwaZulu-Natal College of Health Sciences Durban, ZA, Optics and Imaging Centre<br>Kasambala, Maritha ; University of KwaZulu-Natal Faculty of Science and Agriculture<br>Midzi, Herald; University of KwaZulu-Natal College of Health Sciences Durban, ZA, Optics and Imaging Centre<br>Mduluza-Jokonya, Tariro; University of KwaZulu-Natal College of Health Sciences Durban, ZA, Optics and Imaging Centre<br>Rusakaniko, Simbarashe; University of Zimbabwe College of Health Sciences<br>Mduluza, Takafira; University of Zimbabwe Faculty of Science, Biochemistry Department

Keywords:	Diagnostic microbiology < INFECTIOUS DISEASES, Tropical medicine < INFECTIOUS DISEASES, STATISTICS & RESEARCH METHODS

SCHOLARONE™
Manuscripts

I, the Submitting Author has the right to grant and does grant on behalf of all authors of the Work (as defined in the below author licence), an exclusive licence and/or a non-exclusive licence for contributions from authors who are: i) UK Crown employees; ii) where BMJ has agreed a CC-BY licence shall apply, and/or iii) in accordance with the terms applicable for US Federal Government officers or employees acting as part of their official duties; on a worldwide, perpetual, irrevocable, royalty-free basis to BMJ Publishing Group Ltd ("BMJ") its licensees and where the relevant Journal is co-owned by BMJ to the co-owners of the Journal, to publish the Work in this journal and any other BMJ products and to exploit all rights, as set out in our licence.

The Submitting Author accepts and understands that any supply made under these terms is made by BMJ to the Submitting Author unless you are acting as an employee on behalf of your employer or a postgraduate student of an affiliated institution which is paying any applicable article publishing charge ("APC") for Open Access articles. Where the Submitting Author wishes to make the Work available on an Open Access basis (and intends to pay the relevant APC), the terms of reuse of such Open Access shall be governed by a Creative Commons licence – details of these licences and which Creative Commons licence will apply to this Work are set out in our licence referred to above.

Other than as permitted in any relevant BMJ Author's Self Archiving Policies, I confirm this Work has not been accepted for publication elsewhere, is not being considered for publication elsewhere and does not duplicate material already published. I confirm all authors consent to publication of this Work and authorise the granting of this licence.

For peer review only

Clinical utility of peptide microarrays in the serodiagnosis and surveillance of neglected tropical diseases in sub-Saharan Africa: Protocol for a diagnostic test accuracy systematic review

Arthur Vengesai[1, 2*], Thajasvarie Naicker[2], Maritha Kasambala[2], Herald Midzi[1], Tariro L. Mduluza-Jokonya[1, 2] Simbarashe Rusakaniko[3] and Takafira Mduluza[1]

[1]Department of Biochemistry, University of Zimbabwe, P.O. Box MP 167, Mt Pleasant, Harare, Zimbabwe.

[2]Optics & Imaging, Doris Duke Medical Research Institute, College of Health Sciences, University of KwaZulu-Natal; KwaZulu-Natal, South Africa.

[3]College of Health Sciences, University of Zimbabwe, Box A178 Mazowe Street Avondale

Email addresses

*Corresponding author (arthurvengesai@gmail.com/+263776292361) 5746 New Canaan Harare

naickera@ukzn.ac.za (Thajasvarie Naicker)

marithakasambala@gmail.com (Maritha Kasambala)
midziherald@gmail.com (Herald Midzi)
tljokonya@gmail.com (Tariro Mduluza)
srusakaniko@gmail.com (Simbarashe Rusakaniko)
tmduluza@yahoo.com (Takafira Mduluza)

For peer review only - http://bmjopen.bmj.com/site/about/guidelines.xhtml

ABSTRACT

Introduction: Neglected tropical diseases tend to cluster in the same poor populations and, to make progress with their control, they will have to be dealt with in an integrated manner. Peptide microarrays may be a solution to these problems, where diagnosis for co-infection can be detected simultaneously using the one tool.

Objectives: A meta-analysis using hierarchical models will be performed to assess the diagnostic accuracy of peptide microarrays for detecting schistosomiasis (*S. mansoni* and *S. haematobium),* soil transmitted helminths (*Trichuris trichiura*, *Ascaris lumbricoides* and *Necator americanus*), trachoma (*Chlamydia trachomatis*) and lymphatic filariasis (*Wuchereria bancrofti*) in people residing in sub-Saharan Africa.

Methods and Analysis: A comprehensive search of the following databases will be performed: Cochrane Infectious Diseases Group Specialized Register, PubMed, EMBASE and The Web of Science. Studies comparing peptide microarrays with a reference standard from a random or consecutive series of patients will be included in the study. Two review authors will independently screen titles and abstracts for relevance, assess full text articles for inclusion and carry out data extraction using a tailored data extraction form. The quality Assessment of Diagnostic Accuracy Studies-2 tool will be used to assess the quality of the selected studies. The bivariate model and the hierarchical summary receiver operating characteristic curve model will be performed to evaluate the diagnostic accuracy of the peptide microarrays. Meta-regression analyses will be performed to investigate heterogeneity across studies.

Ethics and dissemination: There is no requirement for ethical approval because the work will carried out using previously published data, without human beings involvement. Findings will be disseminated through peer-reviewed publication and in conferences presentations

Systematic review registration: The protocol for this review was registered at PROSPERO; registration number CRD42020175145.

Article Summary: Strengths and limitations of this study

- This systematic review protocol follows the Preferred Reporting Items for Systematic Review and Meta-Analysis Protocols guidelines.
- This systematic and meta-analysis review will be the first to explore the diagnostic test accuracy of peptide microarrays.
- The methods described in the review may be applicable to any diagnostics test accuracy systematic and meta-analysis reviews.
- There is the potential for poor quality in the reporting of diagnostics test accuracy studies.

For peer review only

- Excluding articles not published in English may lead to publication bias.

Introduction

Neglected tropical diseases (NTDs) are a group of debilitating communicable diseases that affect over 1.6 billion people globally, 40% of whom live on the African continent [1]. Notably, NTDs affect the world's poorest, most marginalized and remote communities, where access to clean water, sanitation and health care is limited. The impact of NTDs on communities are devastating; they cause severe pain, disabilities, deformities, malnutrition, stunted growth, cognitive impairment, social isolation, and humiliation. These diseases may be fatal, in fact anaemia caused by some NTDs have a direct impact on maternal mortality. Importantly, NTDs have a disruptive impact on the productivity of already unstable economies. They keep children out of school and adults out of work hence trapping poor communities in endless cycles of poverty [1–3].

Sub-Saharan Africa is estimated to account for the following worldwide NTDs proportions; approximately 25%-33% soil-transmitted helminth (STH) infections, more than 33% of the lymphatic filariasis (LF), 50% of trachoma infections, most of the world's cases of schistosomiasis, human African trypanosomiasis, loiasis and onchocerciasis, and all of the world's cases of dracunculiasis and human African trypanosomiasis [4,5]. Different NTDs can occur in the same poor populations [6] (illustrated in **figure 1**). In most regions of sub-Saharan Africa it is not uncommon to find five or more NTDs in one area—the three major soil-transmitted helminth infections, schistosomiasis, LF, onchocerciasis, and trachoma [7]. Zimbabwe a country in Southern Africa is endemic to four of the most common NTDs, lymphatic filariasis, schistosomiasis, STH and trachoma [8–10].

Figure 1: Global overlap of the six most common NTDs [11].

The lack of rapid, accurate, simple-to-use, point-of-care tests for many of the neglected tropical diseases is an important feature for their general neglect and the under-appreciation of their disease burden.

Diagnosis plays a crucial role in the surveillance and detection of infectious diseases including NTDs [12]. However NTDs remain mostly undiagnosed, the reliable identification of parasitic infections requires in-depth training for specimen preparation, and expertise for subsequent microscopic examination, that are unavailable in most rural clinics and remote areas [13, 14]. The

For peer review only - http://bmjopen.bmj.com/site/about/guidelines.xhtml

diagnosis of NTDs, is primarily based on well-established and widely used laboratory techniques, such as the examination of blood, stool and urine samples under a microscope [13]. Schistosomiasis is diagnosed through the microscopic detection of parasite eggs in stool or urine specimens using the kato-katz and urine filtration techniques respectively [15]. The standard method for diagnosing the presence of STHs is by microscopically identifying STHs eggs in a stool sample using the Kato Katz technique or The formal ether concentration technique [16]. The standard method for diagnosing LF active infection is the identification of microfilariae in a blood smear by microscopy [17]. Trachoma diagnosis relies on the use of conjunctival examination for the clinical sign of trachomatous inflammation by trained eye specialist. PCR-based assays for trachroma diagnosis are technically complex and too expensive [18].

While microscopy of parasites is considered as highly specific, its sensitivity depends on the intensity of infection and timing of sample collection. Schistosomiasis eggs tend to be passed at irregular intervals and in small amounts and may not be detected [19]. STHs eggs may be difficult to find in light infections [16], hence concentration techniques are often recommended for diagnosis. The microfilariae that cause LF circulate in the blood at night and blood collection should be done at night to coincide with the appearance of the parasite [17]. For trachoma, conjunctival inflammation may persist in the absence of detectable bacteria, an important limitation to decision making based on clinical exams [18].

Serologic tests provide an alternative to microscopic detection of parasites for the diagnosis of schistosomiaisis, STHs, trachoma and LF. Antibodies and/or antigens detected in blood samples are also indications of *Schistosoma* infections [20]. Patients with active filarial infection typically have elevated levels of antifilarial IgG4 in the blood and these can be detected using serological assays [17]. A dipstick immunoassay based on detection of chlamydial lipopolysaccharide was developed for diagnosis of trachoma [18]. Serologic tests may also be useful in showing exposure to infection and the need for thorough examination, treatment and follow-up in people living in low-transmission areas [20].

Engels and Savioli, (2006) recommended diagnosis of NTDs collectively as a group, as they tend to cluster in the same poor populations and, to make progress with their control, they will have to be dealt with in an integrated manner [6] . Peptide microarrays may be a solution to these problems, where diagnosis for co-infection can be detected simultaneously using the one tool.

A microarray is a multiplexed lab-on-a-chip. Peptide microarrays (collections of short peptides of pathogens immobilised on solid planar supports) are large scale screening systems for simultaneous identification of multiple pathogens from small quantities of serum or plasma and other body fluids

For peer review only - http://bmjopen.bmj.com/site/about/guidelines.xhtml

[21]. These microarrays may be used for the diagnosis and surveillance of NTDs. The principle behind microarray chips involves the use of antibody tests that can determine whether an individual is infected or uninfected. Antibody-protein interactions play a critical role in the humoral immune response. B-cells secrete antibodies, which bind antigens. The specific part of antigens that are recognized by antibodies are called B-cell epitopes. These epitopes may be linear, corresponding to a contiguous amino acid sequence fragment of an antigen, or conformational, in which case residues critical for recognition are not contiguous in the primary sequence, but are in close proximity within the folded protein 3D structure [22].

The Expanded Special Project for Elimination of Neglected Tropical Diseases (ESPEN) works to reduce the burden of 5 targeted NTDs, schistosomiasis, STHs, trachoma, LF and onchocerciasis , which put at an estimated 600 million people at risk in Africa [3]. Box 1 gives a brief description of each disease. Hence the objective of the review is to assess the diagnostic accuracy of peptide microarrays for the detection of schistosomiasis (*S. mansoni, S. haematobium),* STHs (*Trichuris trichiura*, *Ascaris lumbricoides* and *Necator americanus*), lymphatic filariasis (*Wuchereria bancrofti*) and trachoma (*Chlamydia trachomatis*) in people living in NTDs endemic areas. Diagnostic accuracy will be determined by sensitivity, specificity, positive and negative predictive values of each test.

Box 1

Schistosomiasis also known as bilharzia or the snail fever is an acute and chronic water-born parasitic disease, caused by infection with trematodes (blood borne flukes) of the genus *Schistosoma* [23]. There are 2 major forms of schistosomiasis – intestinal and urogenital – caused by 5 main species of blood fluke.

Schistosomiasis affects almost 240 million people worldwide, and more than 700 million people live in endemic areas. Globally there are about 436 million people at risk of infection and 112 million people infected with *Shistosoma haematobium* (Urogenital schistosomiasis). *Shistosoma mansoni* the main cause of intestinal schistosomiasis in Sub Saharan Africa puts at risk of infection 393 million people and infects 54 million people globally. Urogenital schistosomiasis is also considered to be a risk factor for HIV infection,

For peer review only - http://bmjopen.bmj.com/site/about/guidelines.xhtml

especially in women. Control measures include mass drug administration, improved sanitation, snail control and avoidance of contact with infested water. The disease is found in tropical and subtropical areas of Asia, Africa and Latin America [15,20].

Soil-transmitted helminth infections (intestinal worms) are among the most common parasitic infections worldwide that affect the poorest and most deprived communities. STHs eggs that are present in faeces of infected humans are transmitted to others. The main species that infect people are *Ascaris lumbricoides* (round worm), *Trichuris trichiura* (whipworm) and the hookworms (*Necator americanus* and *Ancylostoma duodenale*) [24]. A large part of the world's population is infected with one or more of these soil-transmitted helminths: approximately 807-1,121 million, 604-795 million and 576-740 million are infected with *Ascaris lumbrioides*, *Trichuris trichiura* and *Nector americanus* respectively [25]. Infected children are nutritionally and physically impaired [24]. STHs also cause anaemia due to worm induced blood loss and compromised nutrition, intestinal obstruction as well as reduced absorption of vitamin A [9]. STHs can be controlled by periodical deworming campaigns to eliminate infecting worms, health education to prevent re-infection, and improved sanitation practices to reduce soil contamination with infective eggs [24].

Trachoma is the leading infectious cause of blindness worldwide. Infection spreads from person to person, particularly from child to child and from child to mother to child. Trachoma is caused by a contagious bacterium *Chlamydia trachomatis* infection of the eye. Infection spreads through personal contact, commonly through contact with contaminated hands, clothing or bedding and by flies that have been in contact with discharge from the eyes or nose of an infected person. Trachoma disease is usually clinically diagnosed. Trachoma is a public health problem in 44 countries, and is responsible for the blindness or visual impairment of about 1.9 million people. Blindness from trachoma is irreversible. Based on March 2019 data, 142 million people live in trachoma endemic areas and are at risk of trachoma blindness. In 2018, 146 112 people received surgical treatment for advanced stage of the disease, and 89.1 million people were treated with antibiotics [26].

Lymphatic filariasis (LT), commonly known as elephantiasis is a mosquito-transmitted diseases caused by parasitic worms (*Wuchereria bancrofti*, *Brugia* spp.) that damage part

For peer review only - http://bmjopen.bmj.com/site/about/guidelines.xhtml

of the immune system. LT is a painful and profoundly disfiguring disease and can lead to stigma. It is one of the world's leading causes of disability. In communities where filariasis is transmitted, all ages are affected. The painful and profoundly disfiguring visible manifestations of the disease, lymphoedema, elephantiasis and scrotal swelling occur later in life and can lead to permanent disability. In 2000 over 120 million people were infected, with about 40 million disfigured and incapacitated by the disease. Infection is usually acquired in childhood causing hidden damage to the lymphatic system. 893 million people in 49 countries worldwide remain threatened by lymphatic filariasis and require preventive chemotherapy to stop the spread of this parasitic infection. *Wuchereria bancrofti* is responsible for 90% of the case Treatment is through drugs or surgery [17].

Methods

Our methodology for the study will be based on guidelines proposed by the Cochrane Handbook for Systematic Reviews of Diagnostic Accuracy and relevant literature [27–29]. The study will also use the Preferred Reporting Items for Systematic Review and Meta-Analysis (PRISMA) guidelines published by Moher, Liberati to search and select the studies to be included in the systematic review [30]. The PRISMA checklist (Additional file 1) will be used to ensure the inclusion of relevant information in the analysis and the quality assurance appraisal will be used as the basis for literature selection

Additional file 1

Word doc

PRISMA-P Checklist

Search strategy

Electronic searches

A comprehensive search of the literature will done in the general data bases PubMed, EMBASE and The web of science and Cochrane Infectious Diseases Group Specialized Register. The search will be between the inception of each data base to present day, using the search terms and Boolean operators shown in **tables 1, 2, 3** and **4**. Alternative terms and concepts which address the same question will be used as it is common for a range of terms to be used to describe the same

For peer review only - http://bmjopen.bmj.com/site/about/guidelines.xhtml

phenomenon or research area. **Figure 2** is showing the initial search for *S. haematobium* in PubMed. After removing duplicates, articles will be assessed for inclusion into the review through title and abstract analysis. Shortlisted articles will be screened for inclusion through full-text analysis.

Figure 2: Showing the search strategy for *S. haematobium* in PubMed

Table 1: Search strategy for schistosomiasis

Microarray<br>OR<br>Biochip<br>OR<br>Microchip<br>0R<br>Micro chip<br>OR<br>Microarray<br>OR<br>Chip<br>OR<br>Array	AND	Epitope<br>OR<br>Peptide	AND	Schistosomiasis<br>OR<br>Bilharzia<br>OR<br>*S. haematobium*<br>OR<br>*S. mansoni*<br>OR<br>*Schistosoma haematobium*<br>OR<br>*Schistosoma mansoni*

For peer review only

Table 2: Search strategy for soil transmitted helminths

Microarray<br>OR<br>Biochip<br>OR<br>Microchip<br>0R<br>Micro chip<br>OR<br>Microarray	AND	Epitope<br>OR<br>Peptide	AND	Roundworms<br>OR<br>hookworm<br>OR<br>whipworm<br>OR<br>*A. lumbricoides*<br>OR<br>*Nector americanus*

For peer review only - http://bmjopen.bmj.com/site/about/guidelines.xhtml

OR<br>Chip<br>OR<br>Array				OR<br>*Trichuris trichiura*

Table 3: Search strategy for Trachoma

Microarray<br>OR<br>Biochip<br>OR<br>Microchip<br>0R<br>Micro chip<br>OR<br>Microarray<br>OR<br>Chip<br>OR<br>Array	AND	Epitope<br>OR<br>Peptide	AND	Trachoma<br>OR<br>Blinding<br>trachoma<br>OR<br>Granular<br>conjunctivitis<br>OR<br>Egyptain<br>opthalmia<br>OR<br>*Chlamydia*<br>*trachomatis*

Table 4: Search strategy for lymphatic filariasis

Microarray<br>OR<br>Biochip<br>OR<br>Microchip<br>0R<br>Micro chip<br>OR<br>Microarray<br>OR<br>Chip<br>OR	AND	Epitope<br>OR<br>Peptide	AND	Lymphatic<br>filariasis<br>OR<br>elephantiasis<br>OR<br>*Wuchereria*<br>*bancrofti*

For peer review only

Array				

Searching other resources

We will search the proceedings and abstracts of relevant conferences. We will hand search the reference lists of all the relevant primary studies for additional relevant publications for inclusion in the study. We will also search for related articles to the included studies using the PubMed "similar articles" function. PEPperPRINT Heidelberg Germany and other companies involved in the manufacture of peptide microarrays will be contacted to obtain additional unpublished literature from their collaborators.

For peer review only

Criteria for considering studies for this review

Types of studies

Studies evaluating diagnostic test accuracy of peptide microarrays in a consecutive series of patients, or a randomly-selected series of patients, will be eligible. Where the report does not explicitly state that sampling was consecutive, but consecutive sampling was judged most probable, the study will be included. Studies will be excluded if they do not present sufficient data to allow us to (a) extract or calculate absolute numbers of true positives, false positives, false negatives, and true negatives and (b) they provided insufficient information to calculate sensitivity and specificity. Studies will also be excluded if they were not available in English, or if they presented insufficient information to fully assess their eligibility. Review articles, studies of retrospective design where investigators collected samples after execution of the reference test, abstracts of meetings and case reports where full text is not available will be excluded from the study.

Participants

Studies recruiting individuals living in NTDs endemic areas will be included in the study. Studies will be excluded if participants had been treated for the target conditions and the tests would have been performed to assess the treatment outcomes. In studies with broader inclusion criteria but which presented results stratified by subgroups, we will include the data relevant to our inclusion criteria.

Index tests

Studies evaluating all peptide microarray diagnostic tools designed to detect the target conditions in parallel (multiplex diagnostic tool), or individually will be included in the study. Additionally,

For peer review only - http://bmjopen.bmj.com/site/about/guidelines.xhtml

studies that exclusively used full-length proteins for microarray immunoassays will also be included in study

Comparator tests

Studies will be included regardless of whether they made comparisons with other microarray or serological tests.

Target conditions

Studies aimed to detect schistosomiasis (*S. mansoni, S. haematobium),* STHs (*Trichuris trichiura*, *Ascaris lumbricoides* and *Necator americanus*), lymphatic filariasis (*Wuchereria bancrofti*) and trachoma (*Chlamydia trachomatis)* either in parallel or individually will be included in this review article.

Reference standards

Studies that would have used the gold standard diagnostic method of each disease as the reference standard (table 5) will be included in the study.

Table 5: Selected NTDs and their gold standard diagnostic tests

Disease/pathogen	Reference standard
S. haematobium	Microscopic detection of parasite eggs present in urine using the urine filtration. [31]
S. mansoni	Microscopic detection of parasite eggs present in stool using the Kato Katz techniques. [31] Formal ether concentration method and the point-of-care Circulating Cathodic Antigen (POC-CCA) will be considered as alternative reference standards.
STHs (*Trichuris trichiura*, *Ascaris lumbricoides* and *Necator americanus*)	Microscopic detection of parasite eggs present in stool using the Kato Katz techniques. [31] The formal ether concentration technique will be considered as an alternative reference standard.
Wucheria bancrofti	Microscopic examination of microfilariae in a blood smear. [17]

For peer review only - http://bmjopen.bmj.com/site/about/guidelines.xhtml

Trachoma	Blinding will be diagnosed clinically using the WHO grading system for trachoma. [32].

Data collection and analysis

Selection of studies

Two authors will independently assess the titles of studies identified by the search, excluding those obviously irrelevant to the diagnosis of NTDs using microarrays. A single failed eligibility criterion will be sufficient for a study to be excluded from the review study. Letters, review articles, and articles clearly irrelevant based on examination of the abstract and other notes will be excluded next and the eligibility of the remaining potentially relevant articles will be judged on full text publications.

Data extraction (selection and coding)

Campbell *et al* (2015), using standards for the reporting of diagnostic accuracy studies (STARD) checklist developed the data extraction tool that will be used in this review [33]. The following data will be extracted.

- Study authors, publication year, and journal.
- Study design.
- Case definition
- Study participants—age, sex.
- Prevalence of target condition.
- Reference standard (including criteria for positive test).
- Index tests (cut-off values (pre-specified or not) and whether the test was a commercial of in-house test).
- Geographical location of data collection
- Index/reference time interval (Treatment status of participants before study or post treatment).
- Distribution of severity of disease in those with target condition
- Other diagnoses in those without target condition
- Infection intensity (egg counts in urine and stool by microscopy).
- Presence of missing or unavailable test results.
- Numbers of TPs, FNs, FPs, and FNs.

For peer review only - http://bmjopen.bmj.com/site/about/guidelines.xhtml

Data extraction will be conducted independently by two authors (AV and HM) to avoid bias. Any discrepancy will resolved by discussion. Where an agreement cannot be reached, a third author will be consulted. Where it remains unclear whether a study is eligible for inclusion, it will be excluded.

For each comparison of index test with reference test, data will be extracted on the number of true positives, true negatives, false positives and false negatives in the form of a two by two table. Like all studies of diagnostic test accuracy that comply with the STARD statement, the two by two tables will further be used to calculate sensitivities and specificities of the target assays. Where two versions of one reference standard or index test were used, for example local clinic and expert standard microscopy or field versus laboratory testing, only the one most likely to yield the highest quality results was included in the review. In cases of studies where only a subgroup of participants met the review inclusion criteria, data will extracted and presented only for that particular subgroup.

Quality of the studies (Risk of bias assessment)

In order to reduce bias two reviewers will independently assess the quality of each individual study. In cases of disagreements a third reviewer will be consulted in order to resolve the disagreement. The most widely used tool for examining diagnostic test accuracy QUADAS-2 tool (Quality Assessment of Diagnostic Accuracy Studies-2) will be used to assess the quality of studies that will be included. Critical appraisal questions on the checklist will be answered with a yes/no response or will be marked unclear if insufficient information was reported to allow judgement to be made [33,34].

Statistical analysis

We will stratify all diagnostic tests by the target condition and reference standards used. For each study within each stratum, we will construct two-by-two tables for true positives, true negatives, false positives and false negatives. We will construct forest plots displaying sensitivity and specificity of the index test from contingency tables assuming that the reference methods will be 100% sensitive and specific. Where only sensitivity and specificity estimates are reported, we will derive the two-by-two table from the reported data. We will enter the two-by-two data into Review Manager (RevMan) software for Windows (Cochrane Collaboration, Copenhagen, Denmark). Estimates of sensitivity and specificity from each individual study will be summarized on forest plots at 95 % confidence interval and plotted using summary receiver operating characteristic (SROC) plots.

Meta-analysis will be performed in cases where three or more studies are available for the index test and target condition. Studies will also be submitted to meta-analysis when three conditions are met:

For peer review only - http://bmjopen.bmj.com/site/about/guidelines.xhtml

sample size is greater than 20; sensitivity and specificity are available for the index; and when a control group is included in the analysis. Hierarchical/multi-level random models including hierarchical summary receiver operating characteristic model (HSROC) model and the bivariate random effects model, will be used in our study to evaluate the diagnostic accuracy of peptide microarrays. These models allows for both within and between study heterogeneity [35]**.** The bivariate model, models sensitivity and specificity directly at a common threshold and its primary objective is to obtain a summary estimate of sensitivity and specificity. It is recommended for purely binary test or when different studies report similar thresholds [36–38]. HSROC includes a random-effects term for variation in accuracy and threshold between studies, and non-symmetrical underlying ROC curves. This model estimates the underlying ROC curve, which describes how sensitivity and specificity of the included studies trade off with each other as thresholds vary. The position of the ROC curve depends on the degree of overlap of the distributions of the diseased and non-diseased and helps to estimate the level of discriminatory power of a test. The closer the ROC curve is to the upper-left corner of the graph, the better the tests discriminates between diseased and non-diseased. In general, the HSROC model is recommended for continuous tests when the included studies all report a different threshold for test positivity will be used if data from a test has multiple thresholds [35,37].

In this review we will use the bivariate model when data from a test had one or a common threshold to perform the overall meta-analysis. In scenarios where there is between-study variation in thresholds, we will perform meta-analyses by using the HSROC model to estimate SROC curves of sensitivity and specificity. The summary estimates of sensitivity and specificity and 95% CI and the HSROC will calculated using MetaDTA (an interactive web-based tool to conduct and interrogate meta-analysis of diagnostic test accuracy studies) [39].

Assessment of Publication bias

In this review the Begg's rank correlation test [40] and the Egger's regression test [41] will be used to evaluate possible publication bias. Publication bias will be assessed when 5 or more studied are available,

Investigations of heterogeneity

We will visually inspect forest plots and summary ROC plots to investigate the heterogeneity between study specific estimates of sensitivity and specificity. When five or more studies are available we will perform meta-regression analysis to explore the heterogeneity between studies. We will investigate heterogeneity by adding covariates (including, age group, gender, infection intensity and country) in the bivariate model which allows for separate calculations of sensitivity

For peer review only - http://bmjopen.bmj.com/site/about/guidelines.xhtml

and specificity and of the effect of each of the covariates on summary results of sensitivity and specificity. Meta-regression analysis will be performed in OpenMeta-Analyst for Windows 10 (open-source, cross-platform for advanced meta-analysis).

Patient and public involvement

There will be no patient or public involvement in conducting this review article because it will be based on previously published data.

Ethics and dissemination

This diagnostics test accuracy systematic review is exempt from ethical approvals because the work will carried out using previously published data. Findings of the review will be disseminated in an open access peer-reviewed journal and at conferences presentations. The review will also contribute to a PhD degree.

Discussion

Serological assays commonly used for the diagnosis of infectious diseases such as dengue infection are relatively inexpensive and easy to perform compared to biochemical tests, cultures or nucleic acid-based methods (Bergamaschi *et al*., 2019). In cases where faecal specimens are unavailable serology can have a role in diagnosis of STHs and *S. mansoni* [14]. However, for sero-diagnosis of human helminthic infections, many tests in use rely on native antigens, either extracted from whole pathogens maintained in laboratory animals, or cultivated *in vitro* to obtain metabolic antigens. These natural antigens are limited in availability and suffer from batch-to-batch variation and their production is laborious. Recombinant antigens used in sero-diagnostic tests require a high degree of purification to avoid cross-reactivity due to contaminants from the expression system [42]. The limitations associated with native antigens and recombinant antigens such as unspecific binding cross-reactivity in serological diagnosis of various diseases, may be resolved by the use of standardized and highly pure synthetic peptide microarrays [42]. Studies on leishmaniasis, Chagas disease, schistosomiasis, paracoccidioidomycosis, tuberculosis, and, more recently, on cryptococcosis, among others, have shown that this approach has potential for the early diagnosis of the disease, thus reducing the morbi-lethality of individuals affected by these infections and ultimately changing their prognosis [43,44].

For improving diagnostic test performance, it is desirable to identify highly specific and highly reactive epitopes from the proteome of the pathogen in question and synthetically produce the

For peer review only - http://bmjopen.bmj.com/site/about/guidelines.xhtml

corresponding peptide antigens. Synthetic peptides are advantageous for diagnostic applications since they are well defined, easily produced in large amounts, highly pure and often cost-saving if compared to the production of natural antigen in animal models or *in vitro* culture [42].

Also in light of the fact that many cases of infectious diseases are still under-reported or misclassified, peptide microarray analysis opens up new perspectives for the use of antibodies as diagnostic biomarkers for disease surveillance and provides unique access to a more differentiated serological diagnosis [45]. Chase *et al* (2012) demonstrated the feasibility of a fundamentally different approach to diagnosis, purposefully aligned with the concept of proactive health monitoring. They achieved this by measuring the profile of the circulating antibodies, referred to as an immunosignature with a large peptide array of different non-natural sequence peptides [46].

In this context, the availability of reliable and cheap peptide microarrays for the sero-diagnosis of infectious diseases even in low-resource settings is a pressing necessity. Hence the present will conduct a systematic review that focuses on the latest applications of peptide microarrays for the serologic medical diagnosis and surveillance of infectious diseases currently in use globally.

List of Abbreviations

ESPEN	Expanded Special Project for Elimination of Neglected Tropical Diseases.
NTDs	Neglected tropical diseases
STH	Soil transmitted helminths
STARD	Standards for the reporting of diagnostic accuracy studies
HSROC	Hierarchical summary receiver operating characteristic
DTA	Diagnostic test accuracy
PRISMA-DTA	Preferred Reporting Items for Systematic Reviews and Meta-Analyses of Diagnostic test accuracy
QUADAS-2	Quality Assessment of Diagnostic Accuracy Studies-2

Acknowledgements

The authors would like to acknowledge the valuable input of Professor Francisca Mutapi.

References

1. ZIMBABWE.

For peer review only - http://bmjopen.bmj.com/site/about/guidelines.xhtml

For peer review only

2. CDC's Neglected Tropical Diseases Program [Internet]. [cited 2020 Mar 3]. Available from: www.cdc.gov

3. Neglected Tropical Diseases can be eliminated in our lifetime: A Call for Action | WHO | Regional Office for Africa [Internet]. [cited 2020 Mar 3]. Available from: https://www.afro.who.int/health-topics/ntds/ntds-can-be-eliminated

4. Hotez PJ, Asojo OA, Adesina AM. Nigeria: "Ground Zero" for the High Prevalence Neglected Tropical Diseases. PLoS Negl Trop Dis [Internet]. 2012 Jul 31 [cited 2020 Jun 20];6(7):e1600. Available from: https://dx.plos.org/10.1371/journal.pntd.0001600

5. Hotez PJ, Kamath A. Neglected tropical diseases in sub-Saharan Africa: Review of their prevalence, distribution, and disease burden. Vol. 3, PLoS Neglected Tropical Diseases. PLoS Negl Trop Dis; 2009.

6. D E, L S. Reconsidering the Underestimated Burden Caused by Neglected Tropical Diseases. Trends Parasitol. 2006;22(8).

7. Molyneux DH, Hotez PJ, Fenwick A. "Rapid-impact interventions": How a policy of integrated control for Africa's neglected tropical diseases could benefit the poor. Vol. 2, PLoS Medicine. PLoS Med; 2005. p. 1064–70.

8. Zimbabwe | ESPEN [Internet]. [cited 2020 Mar 3]. Available from: http://espen.afro.who.int/countries/zimbabwe

9. Midzi N, Mduluza T, Chimbari MJ, Tshuma C, Charimari L, Mhlanga G, et al. Distribution of Schistosomiasis and Soil Transmitted Helminthiasis in Zimbabwe: Towards a National Plan of Action for Control and Elimination. Kabatereine NB, editor. PLoS Negl Trop Dis [Internet]. 2014 Aug 14 [cited 2020 Mar 3];8(8):e3014. Available from: http://dx.plos.org/10.1371/journal.pntd.0003014

10. Phiri I, Manangazira P, Macleod CK, Mduluza T, Dhobbie T, Chaora SG, et al. The Burden of and Risk Factors for Trachoma in Selected Districts of Zimbabwe: Results of 16 Population-Based Prevalence Surveys. Ophthalmic Epidemiol [Internet]. 2018 Dec 28 [cited 2020 Mar 3];25(sup1):181–91. Available from: http://www.ncbi.nlm.nih.gov/pubmed/28532208

11. Bhutta ZA, Sommerfeld J, Lassi ZS, Salam RA, Das JK. Global burden, distribution, and interventions for infectious diseases of poverty. Infect Dis Poverty [Internet]. 2014 Jul 31 [cited 2020 Jun 5];3(1):21. Available from: https://idpjournal.biomedcentral.com/articles/10.1186/2049-9957-3-21

12. Rydevik G, Innocent GT, McKendrick IJ. Evaluating Diagnostic Tests With Near-Perfect Specificity: Use of a Hui–Walter Approach When Designing a Trial of a DIVA Test for Bovine Tuberculosis. Front Vet Sci [Internet]. 2018 Aug 15 [cited 2020 Feb 26];5(AUG):192. Available from: https://www.frontiersin.org/article/10.3389/fvets.2018.00192/full

13. Utzinger J, Becker SL, Knopp S, Blum J, Neumayr AL, Keiser J, et al. Neglected tropical diseases: Diagnosis, clinical management, treatment and control. Vol. 142, Swiss Medical Weekly. EMH Media; 2012.

14. Khurana S, Sethi S. Laboratory diagnosis of soil transmitted helminthiasis. Trop Parasitol. 2017;7(2):86.

15. WHO | World Health Organization [Internet]. [cited 2020 Jun 5]. Available from:

For peer review only - http://bmjopen.bmj.com/site/about/guidelines.xhtml

https://www.who.int/schistosomiasis/epidemiology/table/en/

16. CDC - Trichuriasis - Diagnosis [Internet]. [cited 2020 Jun 5]. Available from: https://www.cdc.gov/parasites/whipworm/diagnosis.html

17. Lymphatic filariasis [Internet]. [cited 2020 Jun 5]. Available from: https://www.who.int/news-room/fact-sheets/detail/lymphatic-filariasis

18. Lammie P, Solomon A, Secor E, Peeling R. DIAGNOSTIC NEEDS FOR NTD PROGRAMS. 2011;

19. CDC - Schistosomiasis - Diagnosis [Internet]. [cited 2020 Jun 5]. Available from: https://www.cdc.gov/parasites/schistosomiasis/diagnosis.html

20. Schistosomiasis [Internet]. [cited 2020 Jun 5]. Available from: https://www.who.int/news-room/fact-sheets/detail/schistosomiasis

21. Zandian A, Forsström B, Häggmark-Månberg A, Schwenk JM, Uhlén M, Nilsson P, et al. Whole-Proteome Peptide Microarrays for Profiling Autoantibody Repertoires within Multiple Sclerosis and Narcolepsy. J Proteome Res [Internet]. 2017 Mar 3 [cited 2020 Feb 26];16(3):1300–14. Available from: http://www.ncbi.nlm.nih.gov/pubmed/28121444

22. El-Manzalawy Y, Dobbs D, Honavar VG. In silico prediction of linear B-cell epitopes on proteins. In: Methods in Molecular Biology. Humana Press Inc.; 2017. p. 255–64.

23. Colley DG, Bustinduy AL, Secor WE, King CH. Human schistosomiasis. In: The Lancet. Lancet Publishing Group; 2014. p. 2253–64.

24. WHO | Soil-transmitted helminthiases [Internet]. [cited 2020 Jun 5]. Available from: https://www.who.int/gho/neglected_diseases/soil_transmitted_helminthiases/en/

25. CDC - Soil-transmitted Helminths [Internet]. [cited 2020 Jun 5]. Available from: https://www.cdc.gov/parasites/sth/index.html

26. Trachoma [Internet]. [cited 2020 Jun 5]. Available from: https://www.who.int/news-room/fact-sheets/detail/trachoma

27. Handbook for DTA Reviews | Cochrane Screening and Diagnostic Tests [Internet]. [cited 2020 Mar 3]. Available from: https://methods.cochrane.org/sdt/handbook-dta-reviews

28. Abba K, Deeks JJ, Olliaro P, Naing C-M, Jackson SM, Takwoingi Y, et al. Rapid diagnostic tests for diagnosing uncomplicated P. falciparum malaria in endemic countries. Cochrane database Syst Rev [Internet]. 2011 Jul 6 [cited 2020 Mar 3];(7):CD008122. Available from: http://www.ncbi.nlm.nih.gov/pubmed/21735422

29. Huang HS, Tsai CL, Chang J, Hsu TC, Lin S, Lee CC. Multiplex PCR system for the rapid diagnosis of respiratory virus infection: systematic review and meta-analysis. Vol. 24, Clinical Microbiology and Infection. Elsevier B.V.; 2018. p. 1055–63.

30. Moher D, Liberati A, Tetzlaff J, Altman DG, Altman D, Antes G, et al. Preferred reporting items for systematic reviews and meta-analyses: The PRISMA statement. Vol. 151, Annals of Internal Medicine. American College of Physicians; 2009. p. 264–9.

31. George S, Geldhof P, Albonico M, Ame SM, Bethony JM, Engels D, et al. Molecular speciation of soil-transmitted helminths egg isolates collected during six drug efficacy trials in endemic countries. Trans R Soc Trop Med Hyg. 2016;110(11):657.

For peer review only - http://bmjopen.bmj.com/site/about/guidelines.xhtml

32. WHO | What is trachoma [Internet]. [cited 2020 Mar 3]. Available from: https://www.who.int/trachoma/disease/en/

33. Campbell JM, Klugar M, Ding S, Carmody DP, Hakonsen SJ, Jadotte YT, et al. Diagnostic test accuracy: Methods for systematic review and meta-analysis. Int J Evid Based Healthc. 2015 Sep 1;13(3):154–62.

34. Whiting PF, Rutjes AWS, Westwood ME, Mallett S, Deeks JJ, Reitsma JB, et al. QUADAS-2: A Revised Tool for the Quality Assessment of Diagnostic Accuracy Studies [Internet]. 2011 [cited 2020 Mar 3]. Available from: www.annals.org

35. Rutter CM, Gatsonis CA. A hierarchical regression approach to meta-analysis of diagnostic test accuracy evaluations. Stat Med Stat Med. 2001;20:2865–84.

36. Reitsma JB, Glas AS, Rutjes AWS, Scholten RJPM, Bossuyt PM, Zwinderman AH. Bivariate analysis of sensitivity and specificity produces informative summary measures in diagnostic reviews. J Clin Epidemiol. 2005 Oct;58(10):982–90.

37. Macaskill P, Gatsonis C, Deeks J, Harbord R, Takwoingi Y. Cochrane Handbook for Systematic Reviews of Diagnostic Test Accuracy Chapter 10 Analysing and Presenting Results [Internet]. [cited 2020 Jun 20]. Available from: http://srdta.cochrane.org/.

38. Leeflang MMG, Kraaijpoel N. Systematic reviews of diagnostic test accuracy in CMI. Vol. 24, Clinical Microbiology and Infection. Elsevier B.V.; 2018. p. 1115–6.

39. Freeman SC, Kerby CR, Patel A, Cooper NJ, Quinn T, Sutton AJ. Development of an interactive web-based tool to conduct and interrogate meta-analysis of diagnostic test accuracy studies: MetaDTA. BMC Med Res Methodol. 2019 Apr 18;19(1).

40. Operating Characteristics of a Rank Correlation Test for Publication Bias - PubMed [Internet]. [cited 2020 Jun 20]. Available from: https://pubmed.ncbi.nlm.nih.gov/7786990/

41. Egger M, Smith GD, Schneider M, Minder C. Bias in meta-analysis detected by a simple , graphical test measures of funnel plot asymmetry. Bmj [Internet]. 1997 Sep 13 [cited 2020 Jun 20];315(7109):629–34. Available from: http://www.ncbi.nlm.nih.gov/pubmed/9310563%0Ahttp://www.pubmedcentral.nih.gov/articlerender.fcgi?artid=PMC2127453

42. List C, Qi W, Maag E, Gottstein B, Müller N, Felger I. Serodiagnosis of Echinococcus spp. infection: Explorative selection of diagnostic antigens by peptide microarray. PLoS Negl Trop Dis [Internet]. 2010 Aug 3 [cited 2020 Feb 26];4(8):e771. Available from: http://www.ncbi.nlm.nih.gov/pubmed/20689813

43. Noya O, Patarroyo M, Guzman F, de Noya B. Immunodiagnosis of Parasitic Diseases with Synthetic Peptides. Curr Protein Pept Sci [Internet]. 2003 Aug 1 [cited 2020 Mar 3];4(4):299–308. Available from: http://www.eurekaselect.com/openurl/content.php?genre=article&issn=1389-2037&volume=4&issue=4&spage=299

44. Brandão RMS de S, Martins LMS, Monte SJH do. Synthetic Peptides as an Alternative Tool for the Diagnosis of Cryptococcosis. In: Fungal Pathogenicity [Internet]. InTech; 2016 [cited 2020 Feb 26]. Available from: http://www.intechopen.com/books/fungal-pathogenicity/synthetic-peptides-as-an-alternative-tool-for-the-diagnosis-of-cryptococcosis

For peer review only - http://bmjopen.bmj.com/site/about/guidelines.xhtml

45. Grötzinger C. Peptide microarrays for medical applications in autoimmunity, infection, and cancer. In: Methods in Molecular Biology [Internet]. Humana Press Inc.; 2016 [cited 2020 Feb 26]. p. 213–21. Available from: http://www.ncbi.nlm.nih.gov/pubmed/26490478

46. Chase BA, Johnston SA, Legutki JB. Evaluation of biological sample preparation for immunosignature-based diagnostics. Clin Vaccine Immunol. 2012 Mar 1;19(3):352–8.

DECLARATIONS

Ethics approval and consent to participate

Not applicable

Consent for publication

Not applicable

Availability of data and material

Data sets used in the current study is available for sharing after reasonable request.

Conflict of interest

The authors declare that they have no competing interests.

Funding

This research was commissioned by the National Institute for Health Research (NIHR) Global Health Research programme (16/136/33) using UK aid from the UK Government. The views expressed in this publication are those of the author(s) and not necessarily those of the NIHR or the Department of Health and Social Care.

This publication was also supported by a grant from the United States Agency for International Development (USAID) and UK aid from the British people (UK aid) through the Coalition for Operational Research on Neglected Tropical Diseases (COR-NTD) and administered by the African Research Network for Neglected Tropical Diseases (ARNTD). Its contents are solely the responsibility of the author(s) and do not necessarily represent the views of USAID, UK aid, COR-NTD or the ARNTD.

Authors' contributions

AV and TM conceived and designed the study protocol. AV, HM, TM and MK will conduct a comprehensive literature search, screen the literature and extract data. AV will write the review

For peer review only

For peer review only - http://bmjopen.bmj.com/site/about/guidelines.xhtml

paper. TN, SR and TM will provide guidance. All authors will read and approve the final version of the manuscript. All authors have read and approved this protocol.

Word count: 3902 words

For peer review only

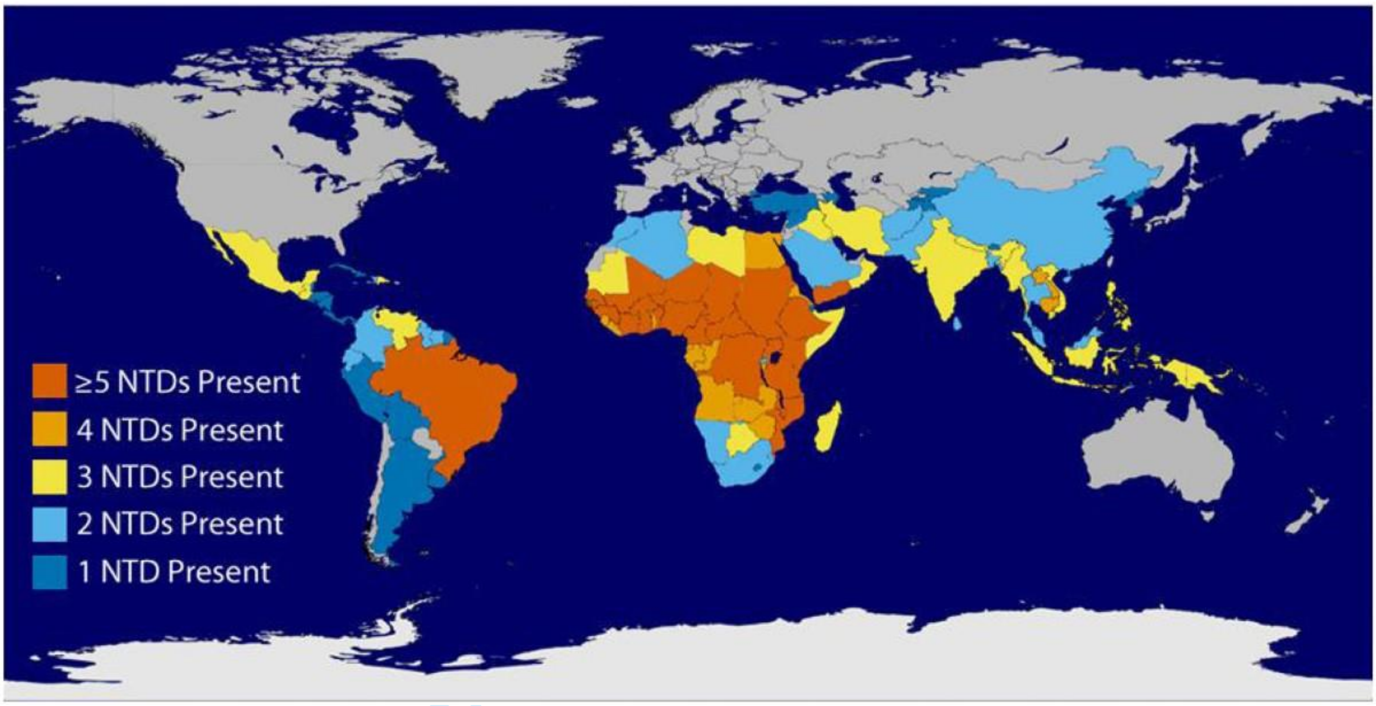

For peer review only

Search	Add to builder	Query	Items found	Time
#16	Add	Search **((((((Microarray Analysis[MeSH Terms]) OR Microarrays) OR Microarray[Title/Abstract]) OR Array[Title/Abstract])) AND ((((Peptides[MeSH Terms]) OR Epitopes[MeSH Terms]) OR Peptide[Title/Abstract]) OR Epitopes[Title/Abstract])) AND ((((Urogenital schistosomiasis) OR Schistosoma haematobium) OR S. haematobium) OR schistosomiasis)**	56	04:53:56
#15	Add	Search **(((Urogenital schistosomiasis) OR Schistosoma haematobium) OR S. haematobium) OR schistosomiasis**	27604	04:53:32
#14	Add	Search **schistosomiasis**	27038	04:53:06
#13	Add	Search **S. haematobium**	2547	04:52:51
#12	Add	Search **Schistosoma haematobium**	3773	04:52:37
#11	Add	Search **Urogenital schistosomiasis**	2548	04:51:59
#10	Add	Search **(((Peptides[MeSH Terms]) OR Epitopes[MeSH Terms]) OR Peptide[Title/Abstract]) OR Epitopes[Title/Abstract]**	2902261	04:51:11
#9	Add	Search **Epitopes[Title/Abstract]**	49899	04:50:47
#8	Add	Search **Peptide[Title/Abstract]**	406530	04:50:16
#7	Add	Search **Epitopes[MeSH Terms]**	111708	04:49:55
#6	Add	Search **Peptides[MeSH Terms]**	2672990	04:49:28
#5	Add	Search **(((Microarray Analysis[MeSH Terms]) OR Microarrays) OR Microarray[Title/Abstract]) OR Array[Title/Abstract]**	275317	04:48:52
#4	Add	Search **Array[Title/Abstract]**	146900	04:46:23
#3	Add	Search **Microarray[Title/Abstract]**	87844	04:45:54
#2	Add	Search **Microarrays**	30663	04:45:28
#1	Add	Search **Microarray Analysis[MeSH Terms]**	91509	04:45:05

For peer review only

PRISMA-P 2015 Checklist

This checklist has been adapted for use with systematic review protocol submissions to BioMed Central journals from Table 3 in Moher D et al: Preferred reporting items for systematic review and meta-analysis protocols (PRISMA-P) 2015 statement. *Systematic Reviews* 2015 **4**:1

An Editorial from the Editors-in-Chief of *Systematic Reviews* details why this checklist was adapted - Moher D, Stewart L & Shekelle P: Implementing PRISMA-P: recommendations for prospective authors. *Systematic Reviews* 2016 **5**:15

Section/topic	#	Checklist item	Information reported		Line number(s)
			Yes	No	
**ADMINISTRATIVE INFORMATION**					
**Title**					
Identification	1a	Identify the report as a protocol of a systematic review	☒	☐	2 and 3
Update	1b	If the protocol is for an update of a previous systematic review, identify as such	☐	☐	N/A
**Registration**	2	If registered, provide the name of the registry (e.g., PROSPERO) and registration number in the Abstract	☒	☐	46 and 47
**Authors**					
Contact	3a	Provide name, institutional affiliation, and e-mail address of all protocol authors; provide physical mailing address of corresponding author	☒	☐	4-18
Contributions	3b	Describe contributions of protocol authors and identify the guarantor of the review	☒	☐	525-529
**Amendments**	4	If the protocol represents an amendment of a previously completed or published protocol, identify as such and list changes; otherwise, state plan for documenting important protocol amendments	☐	☐	N/A
**Support**					
Sources	5a	Indicate sources of financial or other support for the review	☒	☐	514-524
Sponsor	5b	Provide name for the review funder and/or sponsor	☒	☐	514-524
Role of sponsor/funder	5c	Describe roles of funder(s), sponsor(s), and/or institution(s), if any, in developing the protocol	☒	☐	517-518 and 522-524
**INTRODUCTION**					
**Rationale**	6	Describe the rationale for the review in the context of what is already known	☒	☐	117-120

For peer review only

Section/topic	#	Checklist item	Information reported Yes	Information reported No	Line number(s)
**Objectives**	7	Provide an explicit statement of the question(s) the review will address with reference to participants, interventions, comparators, and outcomes (PICO)	☒	☐	136-141
**METHODS**					
**Eligibility criteria**	8	Specify the study characteristics (e.g., PICO, study design, setting, time frame) and report characteristics (e.g., years considered, language, publication status) to be used as criteria for eligibility for the review	☒	☐	189-220
**Information sources**	9	Describe all intended information sources (e.g., electronic databases, contact with study authors, trial registers, or other grey literature sources) with planned dates of coverage	☒	☐	160-163 and 181-186
**Search strategy**	10	Present draft of search strategy to be used for at least one electronic database, including planned limits, such that it could be repeated	☒	☐	165-166 and 169-172
STUDY RECORDS					
Data management	11a	Describe the mechanism(s) that will be used to manage records and data throughout the review	☐	☒	
Selection process	11b	State the process that will be used for selecting studies (e.g., two independent reviewers) through each phase of the review (i.e., screening, eligibility, and inclusion in meta-analysis)	☒	☐	225-230
Data collection process	11c	Describe planned method of extracting data from reports (e.g., piloting forms, done independently, in duplicate), any processes for obtaining and confirming data from investigators	☒	☐	231-262
**Data items**	12	List and define all variables for which data will be sought (e.g., PICO items, funding sources), any pre-planned data assumptions and simplifications	☒	☐	272-273
**Outcomes and prioritization**	13	List and define all outcomes for which data will be sought, including prioritization of main and additional outcomes, with rationale	☒	☐	289-306
**Risk of bias in individual studies**	14	Describe anticipated methods for assessing risk of bias of individual studies, including whether this will be done at the outcome or study level, or both; state how this information will be used in data synthesis	☒	☐	263-269
DATA					
**Synthesis**	15a	Describe criteria under which study data will be quantitatively synthesized	☒	☐	282-285
	15b	If data are appropriate for quantitative synthesis, describe planned summary measures, methods of handling data, and methods of combining data from studies, including any planned exploration of consistency (e.g., I^2, Kendall's tau)	☒	☐	285-288 and 301-306

For peer review only

Section/topic	#	Checklist item	Information reported		Line number(s)
			Yes	No	
	15c	Describe any proposed additional analyses (e.g., sensitivity or subgroup analyses, meta-regression)	☒	☐	312-319
	15d	If quantitative synthesis is not appropriate, describe the type of summary planned	☒	☐	271-281
**Meta-bias(es)**	16	Specify any planned assessment of meta-bias(es) (e.g., publication bias across studies, selective reporting within studies)	☒	☐	307-310
**Confidence in cumulative evidence**	17	Describe how the strength of the body of evidence will be assessed (e.g., GRADE)	☒	☐	264-269

For peer review only

BMJ Open

Clinical utility of peptide microarrays in the serodiagnosis of neglected tropical diseases in sub-Saharan Africa: Protocol for a diagnostic test accuracy systematic review

Journal:	*BMJ Open*
Manuscript ID	bmjopen-2020-042279.R1
Article Type:	Protocol
Date Submitted by the Author:	27-Apr-2021
Complete List of Authors:	Vengesai, Arthur; University of Zimbabwe Faculty of Science, Biochemistry; University of KwaZulu-Natal College of Health Sciences<br>Naicker, Thajasvarie; University of KwaZulu-Natal College of Health Sciences<br>Kasambala, Maritha ; University of KwaZulu-Natal Faculty of Science and Agriculture<br>Midzi, Herald; University of KwaZulu-Natal College of Health Sciences<br>Mduluza-Jokonya, Tariro; University of KwaZulu-Natal College of Health Sciences<br>Rusakaniko, Simbarashe; University of Zimbabwe College of Health Sciences<br>Mduluza, Takafira; University of Zimbabwe Faculty of Science, Biochemistry Department
**Primary Subject Heading**:	Research methods
Secondary Subject Heading:	Diagnostics, Immunology (including allergy), Infectious diseases
Keywords:	Diagnostic microbiology < INFECTIOUS DISEASES, Tropical medicine < INFECTIOUS DISEASES, STATISTICS & RESEARCH METHODS, Infectious diseases & infestations < DERMATOLOGY, Protocols & guidelines < HEALTH SERVICES ADMINISTRATION & MANAGEMENT, Biochemistry < NATURAL SCIENCE DISCIPLINES

SCHOLARONE™
Manuscripts

I, the Submitting Author has the right to grant and does grant on behalf of all authors of the Work (as defined in the below author licence), an exclusive licence and/or a non-exclusive licence for contributions from authors who are: i) UK Crown employees; ii) where BMJ has agreed a CC-BY licence shall apply, and/or iii) in accordance with the terms applicable for US Federal Government officers or employees acting as part of their official duties; on a worldwide, perpetual, irrevocable, royalty-free basis to BMJ Publishing Group Ltd ("BMJ") its licensees and where the relevant Journal is co-owned by BMJ to the co-owners of the Journal, to publish the Work in this journal and any other BMJ products and to exploit all rights, as set out in our licence.

The Submitting Author accepts and understands that any supply made under these terms is made by BMJ to the Submitting Author unless you are acting as an employee on behalf of your employer or a postgraduate student of an affiliated institution which is paying any applicable article publishing charge ("APC") for Open Access articles. Where the Submitting Author wishes to make the Work available on an Open Access basis (and intends to pay the relevant APC), the terms of reuse of such Open Access shall be governed by a Creative Commons licence – details of these licences and which Creative Commons licence will apply to this Work are set out in our licence referred to above.

Other than as permitted in any relevant BMJ Author's Self Archiving Policies, I confirm this Work has not been accepted for publication elsewhere, is not being considered for publication elsewhere and does not duplicate material already published. I confirm all authors consent to publication of this Work and authorise the granting of this licence.

For peer review only

Clinical utility of peptide microarrays in the serodiagnosis of neglected tropical diseases in sub-Saharan Africa: Protocol for a diagnostic test accuracy systematic review

Arthur Vengesai[1, 2*], Thajasvarie Naicker[2], Maritha Kasambala[3], Herald Midzi[1], Tariro L. Mduluza-Jokonya[1, 2] Simbarashe Rusakaniko[4] and Takafira Mduluza[1]

[1]Department of Biochemistry, University of Zimbabwe, P.O. Box MP 167, Mt Pleasant, Harare, Zimbabwe.

[2] University of KwaZulu-Natal College of Health Sciences Durban, ZA

[3]University of KwaZulu-Natal Faculty of Science and Agriculture Durban, ZA

[4]College of Health Sciences, University of Zimbabwe, Box A178 Mazowe Street Avondale

Email addresses

*Corresponding author (arthurvengesai@gmail.com/+263776292361) 5746 New Canaan Harare

naicker@ukzn.ac.za (Thajasvarie Naicker)
marithakasambala@gmail.com (Maritha Kasambala)
midziherald@gmail.com (Herald Midzi)
tljokonya@gmail.com (Tariro Mduluza)
srusakaniko@gmail.com (Simbarashe Rusakaniko)
tmduluza@yahoo.com (Takafira Mduluza)

For peer review only

For peer review only - http://bmjopen.bmj.com/site/about/guidelines.xhtml

ABSTRACT

Introduction: Neglected tropical diseases tend to cluster in the same poor populations and, to make progress with their control, they will have to be dealt with in an integrated manner. Peptide microarrays may be a solution to these problems, where diagnosis for co-infection can be detected simultaneously using the one tool. A meta-analysis using hierarchical models will be performed to assess the diagnostic accuracy of peptide microarrays for detecting schistosomiasis (*S. mansoni* and *S. haematobium),* soil transmitted helminths (*Trichuris trichiura*, *Ascaris lumbricoides* and *Necator americanus*), trachoma (*Chlamydia trachomatis*), lymphatic filariasis (*Wuchereria bancrofti*) and onchocerciasis (*Onchocerca volvulus*) in people residing in sub-Saharan Africa.

Methods and Analysis: A comprehensive search of the following databases will be performed: Cochrane Infectious Diseases Group Specialized Register, PubMed, EMBASE and The Web of Science. Studies comparing peptide microarrays with a reference standard from a random or consecutive series of patients will be included in the study. Two review authors will independently screen titles and abstracts for relevance, assess full text articles for inclusion and carry out data extraction using a tailored data extraction form. The quality Assessment of Diagnostic Accuracy Studies-2 tool will be used to assess the quality of the selected studies. The bivariate model and the hierarchical summary receiver operating characteristic curve model will be performed to evaluate the diagnostic accuracy of the peptide microarrays. Meta-regression analyses will be performed to investigate heterogeneity across studies.

Ethics and dissemination: There is no requirement for ethical approval because the work will be carried out using previously published data, without human beings involvement. Findings will be disseminated through peer-reviewed publication and in conference presentations.

Systematic review registration: The protocol for this review was registered at PROSPERO; registration number CRD42020175145.

Article Summary: Strengths and limitations of this study

- This systematic review protocol follows the Preferred Reporting Items for Systematic Review and Meta-Analysis Protocols guidelines.
- This systematic and meta-analysis review will be the first to explore the diagnostic test accuracy of peptide microarrays.
- The methods described in the review may be applicable to any diagnostics test accuracy systematic and meta-analysis review.
- There is the potential for poor quality in the reporting of diagnostics test accuracy studies.
- Excluding articles not published in English may lead to publication bias.

For peer review only - http://bmjopen.bmj.com/site/about/guidelines.xhtml

For peer review only

Introduction

Neglected tropical diseases (NTDs) are a group of debilitating communicable diseases that affect over 1.6 billion people globally, 40% of whom live on the African continent [1]. Notably, NTDs affect the world's poorest, most marginalized and remote communities, where access to clean water, sanitation and health care is limited. The impact of NTDs on communities are devastating; they cause severe pain, disabilities, deformities, malnutrition, stunted growth, cognitive impairment, social isolation, and humiliation. These diseases may be fatal, in fact anaemia caused by some NTDs have a direct impact on maternal mortality. Importantly, NTDs have a disruptive impact on the productivity of already unstable economies. They keep children out of school and adults out of work hence trapping poor communities in endless cycles of poverty [1–3].

Sub-Saharan Africa is estimated to account for the following worldwide NTDs proportions; approximately 25%-33% of soil-transmitted helminth (STH) infections, more than 33% of the lymphatic filariasis infections, 50% of trachoma infections, most of the world's cases of schistosomiasis, human African trypanosomiasis, loiasis and onchocerciasis, and all of the world's cases of dracunculiasis and human African trypanosomiasis [4,5]. Different NTDs can occur in the same poor populations [6] (illustrated in **Figure 1**). In most regions of sub-Saharan Africa it is not uncommon to find five or more NTDs in one area - the three major soil-transmitted helminth infections, schistosomiasis, lymphatic filariasis, onchocerciasis, and trachoma [7]. Zimbabwe a country in Southern Africa is endemic to four of the most common NTDs, lymphatic filariasis, schistosomiasis, STH and trachoma [8–10].

Figure 1: Global overlap of the six most common NTDs [11].

The lack of rapid, accurate, simple-to-use, point-of-care tests for many of the neglected tropical diseases is an important feature for their general neglect and the under-appreciation of their disease burden.

Diagnosis plays a crucial role in the surveillance and detection of infectious diseases including NTDs [12]. However NTDs remain mostly undiagnosed, the reliable identification of parasitic infections requires in-depth training for specimen preparation, and expertise for subsequent microscopic examination, that are unavailable in most rural clinics and remote areas [13, 14]. The diagnosis of NTDs, is primarily based on well-established and widely used laboratory techniques,

For peer review only - http://bmjopen.bmj.com/site/about/guidelines.xhtml

such as the examination of blood, stool and urine samples under a microscope [13]. Schistosomiasis is diagnosed through the microscopic detection of parasite eggs in stool or urine specimens using the Kato-Katz and urine filtration techniques, respectively [15]. The standard method for diagnosing the presence of STHs is by microscopically identifying STHs eggs in a stool sample using the Kato Katz technique or the formal ether concentration technique [16]. The standard method for diagnosing lymphatic filariasis active infection is the identification of microfilariae in a blood smear by microscopy [17]. Trachoma diagnosis relies on the use of conjunctival examination for the clinical sign of trachomatous inflammation by trained eye specialist. PCR-based assays for trachoma diagnosis are technically complex and too expensive [18]. Microfilariae microscopic examination of skin biopsies (snips) is the gold standard test for diagnosis of onchocerciasis. The biopsy is performed using a sclerocorneal biopsy punch or by elevating a small cone of skin with a needle and slicing it off with a scalpel to obtain bloodless skin [19,20].

While microscopy of parasites is considered as highly specific, its sensitivity depends on the intensity of infection and timing of sample collection. Schistosomiasis eggs tend to be passed at irregular intervals and in small amounts and may not be detected [21]. STHs eggs may be difficult to find in light infections [16], hence concentration techniques are often recommended for diagnosis. The microfilariae that cause lymphatic filariasis circulate in the blood at night and blood collection should be done at night to coincide with the appearance of the parasite [17]. For trachoma, conjunctival inflammation may persist in the absence of detectable bacteria, an important limitation to decision making based on clinical exams [18]. Furthermore, in Loiasis endemic areas the specificity of skin snips for the diagnosis of onchocerciasis is limited. Performing PCR of the skin snip can increase the sensitivity and specificity, however the PCR tests are not commercially available [19,22].

Serologic tests provide an alternative to microscopic detection of parasites for the diagnosis of schistosomiaisis, STHs, trachoma, lymphatic filariasis and onchocerciasis. Antibodies and/or antigens detected in blood samples are also indications of *Schistosoma* infections [23]. Patients with active filarial infection typically have elevated levels of anti-filarial IgG4 in the blood and these can be detected using serological assays [17]. A dipstick immunoassay based on detection of chlamydial lipopolysaccharide was developed for diagnosis of trachoma [18]. Serologic tests may also be useful in showing exposure to infection and the need for thorough examination, treatment and follow-up in people living in low-transmission areas [23].

For peer review only - http://bmjopen.bmj.com/site/about/guidelines.xhtml

Engels and Savioli, (2006) recommended diagnosis of NTDs collectively as a group, as they tend to cluster in the same poor populations and, to make progress with their control, they need to be dealt with in an integrated manner [6] Peptide microarrays may be a solution to these problems, where NTDs co-infections can be diagnosed simultaneously using one sample and tool.

Peptide microarrays (collections of short peptides of pathogens immobilised on solid planar supports) are large scale screening systems for simultaneous identification of multiple pathogens from small quantities of serum or plasma and other body fluids [24]. The principle behind peptide microarrays involves the use of antibody tests that can determine whether an individual is infected or uninfected. Antibody-protein interactions play a critical role in the humoral immune response. B-cells secrete antibodies, which bind antigens. The specific part of antigens that are recognized by antibodies are called B-cell epitopes. These epitopes may be short linear (continuous) peptides, corresponding to contiguous amino acid residues on the surface of an antigen, or conformational (discontinuous), in which case the residues are not sequential in the primary structure, but are in close proximity within the folded protein 3D structure [25,26].

The Expanded Special Project for Elimination of Neglected Tropical Diseases (ESPEN) works to reduce the burden of 5 targeted NTDs, schistosomiasis, STHs, trachoma, LF and onchocerciasis , which put at an estimated 600 million people at risk in Africa [3]. Against this background, the objective of the review is to assess the diagnostic accuracy of peptide microarrays for the detection of schistosomiasis (*S. mansoni, S. haematobium),* STHs (*Trichuris trichiura*, *Ascaris lumbricoides* and *Necator americanus*), lymphatic filariasis (*Wuchereria bancrofti*), trachoma (*Chlamydia trachomatis*) and onchocerciasis (*Onchocerca volvulus*) in people living in endemic areas. Diagnostic accuracy will be determined by sensitivity and specificity of each test.

Methods

Our methodology for the study will be based, on guidelines proposed by the Cochrane Handbook for Systematic Reviews of Diagnostic Accuracy and from relevant literature [27–29]. The study will also use the Preferred Reporting Items for Systematic Review and Meta-Analysis (PRISMA) guidelines published by Moher, Liberati to search and select the studies to be included in the systematic review [30]. The PRISMA checklist (Additional file 1) will be used to ensure the inclusion of relevant information in the analysis and the quality assurance appraisal will be used as the basis for literature selection.

For peer review only - http://bmjopen.bmj.com/site/about/guidelines.xhtml

Additional file 1

Word doc

PRISMA-P Checklist

Search strategy

Electronic searches

A comprehensive search of the literature will done in the general data bases PubMed, EMBASE and the web of science and Cochrane Infectious Diseases Group Specialized Register. The search will be between the inception of each data base to present day, using the search terms and Boolean operators shown in **Tables 1-5**. Alternative terms and concepts which address the same question will be used as it is common for a range of terms to be used to describe the same phenomenon or research area. **Figure 2** is showing the initial search for *S. haematobium* in PubMed. After removing duplicates, articles will be assessed for inclusion into the review through title and abstract analysis. Shortlisted articles will be screened for inclusion through full-text analysis.

Figure 2: Showing the search strategy for *S. haematobium* in PubMed

Table 1: Search strategy for schistosomiasis

Microarray<br>OR<br>Biochip<br>OR<br>Microchip<br>0R<br>Micro chip<br>OR<br>Microarray<br>OR<br>Chip<br>OR<br>Array	AND	Epitope<br>OR<br>Peptide	AND	Schistosomiasis<br>OR<br>Bilharzia<br>OR<br>*S. haematobium*<br>OR<br>*S. mansoni*<br>OR<br>*Schistosoma haematobium*<br>OR<br>*Schistosoma mansoni*

Table 2: Search strategy for soil transmitted helminths

Microarray				Roundworms

For peer review only - http://bmjopen.bmj.com/site/about/guidelines.xhtml

For peer review only

OR<br>Biochip<br>OR<br>Microchip<br>0R<br>Micro chip<br>OR<br>Microarray<br>OR<br>Chip<br>OR<br>Array	AND	Epitope<br>OR<br>Peptide	AND	OR<br>hookworm<br>OR<br>whipworm<br>OR<br>*A. lumbricoides*<br>OR<br>*Nector americanus*<br>OR<br>*Trichuris trichiura*

Table 3: Search strategy for Trachoma

Microarray<br>OR<br>Biochip<br>OR<br>Microchip<br>0R<br>Micro chip<br>OR<br>Microarray<br>OR<br>Chip<br>OR<br>Array	AND	Epitope<br>OR<br>Peptide	AND	Trachoma<br>OR<br>Blinding trachoma<br>OR<br>Granular conjunctivitis<br>OR<br>Egyptain opthalmia<br>OR<br>*Chlamydia trachomatis*

Table 4: Search strategy for lymphatic filariasis

Microarray<br>OR<br>Biochip<br>OR<br>Microchip<br>0R<br>Micro chip<br>OR<br>Microarray<br>OR<br>Chip<br>OR<br>Array	AND	Epitope<br>OR<br>Peptide	AND	Lymphatic filariasis<br>OR<br>elephantiasis<br>OR<br>*Wuchereria bancrofti*

Table 5: Search strategy for onchocerciasis

Microarray				

For peer review only - http://bmjopen.bmj.com/site/about/guidelines.xhtml

OR<br>Biochip<br>OR<br>Microchip<br>0R<br>Micro chip<br>OR<br>Microarray<br>OR<br>Chip<br>OR<br>Array	AND	Epitope<br>OR<br>Peptide	AND	Onchocerciasis<br>OR<br>River blindness<br>OR<br>*Onchocerca volvulus*

Searching other resources

We will search the proceedings and abstracts of relevant conferences. We will hand search the reference lists of all the relevant primary studies for additional relevant publications for inclusion in the study. We will also search for relevant articles using the PubMed "similar articles" function. PEPperPRINT Heidelberg Germany and other companies involved in the manufacture of peptide microarrays will be contacted to obtain additional unpublished literature from their collaborators.

Criteria for considering studies for this review

Types of studies

Studies evaluating diagnostic test accuracy of peptide microarrays in a consecutive series of patients, or a randomly-selected series of patients, will be eligible. Where the report does not explicitly state that sampling was consecutive, but consecutive sampling was judged most probable, the study will be included. Studies will be excluded if they do not present sufficient data to allow us to (a) extract or calculate absolute numbers of true positives, false positives, false negatives, and true negatives and (b) they provided insufficient information to calculate sensitivity and specificity. Studies will also be excluded if they were not available in English, or if they presented insufficient information to fully assess their eligibility. Review articles, studies of retrospective design where investigators collected samples after execution of the reference test, abstracts of meetings and case reports where full text is not available will be excluded from the study.

Participants

Studies recruiting individuals from sub-Saharan Africa will be included in the study. Studies will be excluded if participants had been treated for the target conditions and the tests would have been performed to assess the treatment outcomes. In studies with broader inclusion criteria but which presented results stratified by subgroups, we will include the data relevant to our inclusion criteria.

For peer review only

For peer review only - http://bmjopen.bmj.com/site/about/guidelines.xhtml

Index tests

Studies evaluating all peptide microarray diagnostic tools, designed to detect the target conditions in parallel (multiplex diagnostics), or individually will be included in the study. Additionally, studies that exclusively used full-length proteins for microarray immunoassays will also be included in study

Comparator tests

Studies will be included regardless of whether they made comparisons with other microarray or serological tests.

Target conditions

Studies aimed to detect schistosomiasis (*S. mansoni, S. haematobium),* STHs (*Trichuris trichiura*, *Ascaris lumbricoides* and *Necator americanus*), trachoma (*Chlamydia trachomatis),* lymphatic filariasis (*Wuchereria bancrofti*) and onchocerciasis (*Onchocerca volvulus*) either in parallel or individually will be included in this review article.

Reference standards

Studies that would have used the gold standard diagnostic method of each disease as the reference standard (**Table 6**) will be included in the study.

Table 6: Selected NTDs and their gold standard diagnostic tests

Disease/pathogen	Reference standard
S. haematobium	Microscopic detection of parasite eggs present in urine using the urine filtration [31].
S. mansoni	Microscopic detection of parasite eggs present in stool using the Kato Katz techniques [31]. Formal ether concentration method and the point-of-care Circulating Cathodic Antigen (POC-CCA) will be considered as alternative reference standards.
STHs (*Trichuris trichiura*, *Ascaris lumbricoides* and *Necator americanus*)	Microscopic detection of parasite eggs present in stool using the Kato Katz techniques [31]. The formal ether

For peer review only

	concentration technique will be considered as an alternative reference standard.
Chlamydia trachomatis	Blinding will be diagnosed clinically using the WHO grading system for trachoma [32].
Wucheria bancrofti	Microscopic examination of microfilariae in a blood smear [17].
Onchocerca volvulus	Skin snip biopsy [19].

Data collection and analysis

Selection of studies

Two authors will independently assess the titles of studies identified by the search, excluding those obviously irrelevant to the diagnosis of the target conditions using peptide microarrays. A single failed eligibility criterion will be sufficient for a study to be excluded from the review study. Letters, review articles, and articles clearly irrelevant based on examination of the abstract and other notes will be excluded next and the eligibility of the remaining potentially relevant articles will be judged on full text publications.

Data extraction (selection and coding)

Campbell *et al* (2015), using standards for the reporting of diagnostic accuracy studies (STARD) checklist developed the data extraction tool that will be used in this review [33]. The following data will be extracted.

- Study authors, publication year, and journal.
- Study design.
- Case definition.
- Study participants—age, sex.
- Prevalence of target condition.
- Reference standard (including criteria for positive test).
- Index tests (cut-off values (pre-specified or not) and whether the test was a commercial of in-house test).
- Geographical location of data collection.
- Index/reference time interval (Treatment status of participants before study or post treatment).
- Distribution of severity of disease in those with target condition

For peer review only - http://bmjopen.bmj.com/site/about/guidelines.xhtml

- Other diagnoses in those without target condition.
- Infection intensity (egg counts in urine and stool by microscopy).
- Presence of missing or unavailable test results.
- Numbers of true positives, true negatives, false positives, and false negatives.

Data extraction will be conducted independently by two authors to avoid bias. Any discrepancy will resolved by discussion. Where an agreement cannot be reached, a third author will be consulted. Where it remains unclear whether a study is eligible for inclusion, it will be excluded.

For each comparison of the index test with the reference test, data will be extracted on the number of true positives, true negatives, false positives and false negatives in the form of a two by two table. Like all studies of diagnostic test accuracy that comply with the STARD statement, the two by two tables will further be used to calculate sensitivities and specificities of the target assays. Where two versions of one reference standard or index test are used, for example local clinic and expert standard microscopy or field versus laboratory testing, only the one most likely to yield the highest quality results will be included in the review. In cases of studies where only a subgroup of participants met the review inclusion criteria, data will extracted and presented only for that particular subgroup.

Quality of the studies (Risk of bias assessment)

In order to reduce bias two reviewers will independently assess the quality of each individual study. In cases of disagreements a third reviewer will be consulted in order to resolve the disagreement. The most widely used tool for examining diagnostic test accuracy QUADAS-2 tool (Quality Assessment of Diagnostic Accuracy Studies-2) will be used to assess the quality of studies that will be included. Critical appraisal questions on the checklist will be answered with a yes/no response or will be marked unclear if insufficient information was reported to allow judgement to be made [33,34].

Statistical analysis

We will stratify all diagnostic tests by the target condition and reference standards used. For each study within each stratum, we will construct two-by-two tables for true positives, true negatives, false positives and false negatives. We will construct forest plots displaying sensitivity and specificity of the index test from the contingency tables assuming that the reference methods will be 100% sensitive and specific. Where only sensitivity and specificity estimates are reported, we will derive the two-by-two table from the reported data. We will enter the two-by-two data into Review Manager (RevMan) software for Windows (Cochrane Collaboration, Copenhagen, Denmark).

For peer review only - http://bmjopen.bmj.com/site/about/guidelines.xhtml

Estimates of sensitivity and specificity from each individual study will be summarized on forest plots at 95 % confidence interval and plotted using summary receiver operating characteristic (SROC) plots.

Meta-analysis will be performed in cases where three or more studies are available for the index test and target condition. Studies will also be submitted to meta-analysis when three conditions are met: sample size is greater than 20; sensitivity and specificity are available for the index; and when a control group is included in the analysis. Hierarchical/multi-level random models including hierarchical summary receiver operating characteristic (HSROC) model and the bivariate random effects model, will be used in our study to evaluate the diagnostic accuracy of peptide microarrays. These models allows for both within and between study heterogeneity [35]**.** The bivariate model, models sensitivity and specificity directly at a common threshold and its primary objective is to obtain a summary estimate of sensitivity and specificity. It is recommended for purely binary test or when different studies report similar thresholds [36–38]. HSROC includes a random-effects term for variation in accuracy and threshold between studies, and non-symmetrical underlying ROC curves. This model estimates the underlying ROC curve, which describes how sensitivity and specificity of the included studies trade off with each other as thresholds vary. The position of the ROC curve depends on the degree of overlap of the distributions of the diseased and non-diseased and helps to estimate the level of discriminatory power of a test. The closer the ROC curve is to the upper-left corner of the graph, the better the tests discriminates between diseased and non-diseased. In general, the HSROC model is recommended for continuous tests when the included studies all report a different threshold for test positivity will be used if data from a test has multiple thresholds [35,37].

In this review we will use the bivariate model when data from a test had one or a common threshold to perform the overall meta-analysis. In scenarios where there is between-study variation in thresholds, we will perform meta-analyses by using the HSROC model to estimate SROC curves of sensitivity and specificity. The summary estimates of sensitivity and specificity and 95% CI and the HSROC will calculated using MetaDTA (an interactive web-based tool to conduct and interrogate meta-analysis of diagnostic test accuracy studies) [39].

Assessment of Publication bias

In this review the Begg's rank correlation test [40] and the Egger's regression test [41] will be used to evaluate possible publication bias. Publication bias will be assessed when 5 or more studied are available.

Investigations of heterogeneity

For peer review only - http://bmjopen.bmj.com/site/about/guidelines.xhtml

We will visually inspect forest plots and summary ROC plots to investigate the heterogeneity between study specific estimates of sensitivity and specificity. When five or more studies are available we will perform meta-regression analysis to explore the heterogeneity between studies. We will investigate heterogeneity by adding covariates (including, age group, gender, infection intensity and country) in the bivariate model which allows for separate calculations of sensitivity and specificity and of the effect of each of the covariates on summary results of sensitivity and specificity. Meta-regression analysis will be performed in OpenMeta-Analyst for Windows 10 (open-source, cross-platform for advanced meta-analysis).

Patient and public involvement

There will be no patient or public involvement in conducting this review article because it will be based on previously published data.

Ethics and dissemination

This diagnostics test accuracy systematic review protocol is exempt from ethical approvals because the work will carried out using previously published data. Findings of the review will be disseminated in an open access peer-reviewed journal and at conferences presentations. The review will also contribute to a PhD degree.

Discussion

Serological assays commonly used for the diagnosis of infectious diseases such as dengue infection are relatively inexpensive and easy to perform compared to biochemical tests, cultures or nucleic acid-based methods [42]. In cases where faecal specimens are unavailable serology can have a role in diagnosis of STHs and *S. mansoni* [14]. However, for sero-diagnosis of human helminthic infections, many tests in use rely on native antigens, either extracted from whole pathogens maintained in laboratory animals, or cultivated *in vitro* to obtain metabolic antigens. These natural antigens are limited in availability and suffer from batch-to-batch variation and their production is laborious. Recombinant antigens used in sero-diagnostic tests require a high degree of purification to avoid cross-reactivity due to contaminants from the expression system [43]. The limitations associated with native antigens and recombinant antigens such as unspecific binding and cross-reactivity in serological diagnosis of various diseases, may be resolved by the use of standardized and highly pure synthetic peptide [43]. Studies on leishmaniasis, Chagas disease, schistosomiasis, paracoccidioidomycosis, tuberculosis, and, more recently, on cryptococcosis, among others, have

For peer review only - http://bmjopen.bmj.com/site/about/guidelines.xhtml

shown that this approach has potential for the early diagnosis of disease, thus reducing the morbi-lethality of individuals affected by these infections and ultimately changing their prognosis [44,45].

For improving diagnostic test performance, it is desirable to identify highly specific and highly reactive epitopes from the proteome of the pathogen in question and synthetically produce the corresponding peptide antigens. Synthetic peptides are advantageous for diagnostic applications since they are well defined, easily produced in large amounts, highly pure and often cost-saving if compared to the production of natural antigen in animal models or *in vitro* culture [43].

Also in light of the fact that many cases of infectious diseases are still under-reported or misclassified, peptide microarray analysis opens up new perspectives for the use of antibodies as diagnostic biomarkers for disease surveillance and provides unique access to a more differentiated serological diagnosis [46]. Hence in the present study we will conduct a systematic review that focuses on the latest applications of peptide microarrays in the serologic diagnosis and surveillance of NTDs.

List of Abbreviations

ESPEN	Expanded Special Project for Elimination of Neglected Tropical Diseases.
NTDs	Neglected tropical diseases
STH	Soil transmitted helminths
STARD	Standards for the reporting of diagnostic accuracy studies
HSROC	Hierarchical summary receiver operating characteristic
DTA	Diagnostic test accuracy
PRISMA-DTA	Preferred Reporting Items for Systematic Reviews and Meta-Analyses of Diagnostic test accuracy
QUADAS-2	Quality Assessment of Diagnostic Accuracy Studies-2

Authors' contributions

AV and TM conceived and designed the study protocol. AV, HM, TM and MK will conduct a comprehensive literature search, screen the literature and extract data. AV will write the review paper. TN, SR and TM will provide guidance. All authors will read and approve the final version of the manuscript. All authors have read and approved this protocol.

For peer review only - http://bmjopen.bmj.com/site/about/guidelines.xhtml

Conflict of interest

The authors declare that they have no competing interests.

Funding

This research was commissioned by the National Institute for Health Research (NIHR) Global Health Research programme (16/136/33) using UK aid from the UK Government. The views expressed in this publication are those of the author(s) and not necessarily those of the NIHR or the Department of Health and Social Care.

This publication was also supported by a grant from the United States Agency for International Development (USAID) and UK aid from the British people (UK aid) through the Coalition for Operational Research on Neglected Tropical Diseases (COR-NTD) and administered by the African Research Network for Neglected Tropical Diseases (ARNTD). Its contents are solely the responsibility of the author(s) and do not necessarily represent the views of USAID, UK aid, COR-NTD or the ARNTD. Grant number SGPIII/0210/238.

Acknowledgements

The authors would like to acknowledge the valuable input of Professor Francisca Mutapi.

References

1. ZIMBABWE.
2. CDC's Neglected Tropical Diseases Program [Internet]. [cited 2020 Mar 3]. Available from: www.cdc.gov
3. Neglected Tropical Diseases can be eliminated in our lifetime: A Call for Action | WHO | Regional Office for Africa [Internet]. [cited 2020 Mar 3]. Available from: https://www.afro.who.int/health-topics/ntds/ntds-can-be-eliminated
4. Hotez PJ, Asojo OA, Adesina AM. Nigeria: "Ground Zero" for the High Prevalence Neglected Tropical Diseases. PLoS Negl Trop Dis [Internet]. 2012 Jul 31 [cited 2020 Jun 20];6(7):e1600. Available from: https://dx.plos.org/10.1371/journal.pntd.0001600
5. Hotez PJ, Kamath A. Neglected tropical diseases in sub-Saharan Africa: Review of their prevalence, distribution, and disease burden. Vol. 3, PLoS Neglected Tropical Diseases. PLoS Negl Trop Dis; 2009.
6. D E, L S. Reconsidering the Underestimated Burden Caused by Neglected Tropical Diseases. Trends Parasitol. 2006;22(8).
7. Molyneux DH, Hotez PJ, Fenwick A. "Rapid-impact interventions": How a policy of integrated control for Africa's neglected tropical diseases could benefit the poor. Vol. 2, PLoS Medicine. PLoS Med; 2005. p. 1064–70.

For peer review only - http://bmjopen.bmj.com/site/about/guidelines.xhtml

8. Zimbabwe | ESPEN [Internet]. [cited 2020 Mar 3]. Available from: http://espen.afro.who.int/countries/zimbabwe

9. Midzi N, Mduluza T, Chimbari MJ, Tshuma C, Charimari L, Mhlanga G, et al. Distribution of Schistosomiasis and Soil Transmitted Helminthiasis in Zimbabwe: Towards a National Plan of Action for Control and Elimination. Kabatereine NB, editor. PLoS Negl Trop Dis [Internet]. 2014 Aug 14 [cited 2020 Mar 3];8(8):e3014. Available from: http://dx.plos.org/10.1371/journal.pntd.0003014

10. Phiri I, Manangazira P, Macleod CK, Mduluza T, Dhobbie T, Chaora SG, et al. The Burden of and Risk Factors for Trachoma in Selected Districts of Zimbabwe: Results of 16 Population-Based Prevalence Surveys. Ophthalmic Epidemiol [Internet]. 2018 Dec 28 [cited 2020 Mar 3];25(sup1):181–91. Available from: http://www.ncbi.nlm.nih.gov/pubmed/28532208

11. Bhutta ZA, Sommerfeld J, Lassi ZS, Salam RA, Das JK. Global burden, distribution, and interventions for infectious diseases of poverty. Infect Dis Poverty [Internet]. 2014 Jul 31 [cited 2020 Jun 5];3(1):21. Available from: https://idpjournal.biomedcentral.com/articles/10.1186/2049-9957-3-21

12. Rydevik G, Innocent GT, McKendrick IJ. Evaluating Diagnostic Tests With Near-Perfect Specificity: Use of a Hui–Walter Approach When Designing a Trial of a DIVA Test for Bovine Tuberculosis. Front Vet Sci [Internet]. 2018 Aug 15 [cited 2020 Feb 26];5(AUG):192. Available from: https://www.frontiersin.org/article/10.3389/fvets.2018.00192/full

13. Utzinger J, Becker SL, Knopp S, Blum J, Neumayr AL, Keiser J, et al. Neglected tropical diseases: Diagnosis, clinical management, treatment and control. Vol. 142, Swiss Medical Weekly. EMH Media; 2012.

14. Khurana S, Sethi S. Laboratory diagnosis of soil transmitted helminthiasis. Trop Parasitol. 2017;7(2):86.

15. WHO | World Health Organization [Internet]. [cited 2020 Jun 5]. Available from: https://www.who.int/schistosomiasis/epidemiology/table/en/

16. CDC - Trichuriasis - Diagnosis [Internet]. [cited 2020 Jun 5]. Available from: https://www.cdc.gov/parasites/whipworm/diagnosis.html

17. Lymphatic filariasis [Internet]. [cited 2020 Jun 5]. Available from: https://www.who.int/news-room/fact-sheets/detail/lymphatic-filariasis

18. Lammie P, Solomon A, Secor E, Peeling R. DIAGNOSTIC NEEDS FOR NTD PROGRAMS. 2011;

19. CDC - Onchocerciasis - Resources for Health Professionals [Internet]. [cited 2021 Apr 12]. Available from: https://www.cdc.gov/parasites/onchocerciasis/health_professionals/index.html

20. Unnasch TR, Golden A, Cama V, Cantey PT. Diagnostics for onchocerciasis in the era of elimination [Internet]. Vol. 10, International Health. Oxford University Press; 2018 [cited 2021 Apr 12]. p. i20–6. Available from: /pmc/articles/PMC5881263/

21. CDC - Schistosomiasis - Diagnosis [Internet]. [cited 2020 Jun 5]. Available from: https://www.cdc.gov/parasites/schistosomiasis/diagnosis.html

22. Prevention C-C for DC and. CDC - Loiasis. 2021;

For peer review only

For peer review only - http://bmjopen.bmj.com/site/about/guidelines.xhtml

23. Schistosomiasis [Internet]. [cited 2020 Jun 5]. Available from: https://www.who.int/news-room/fact-sheets/detail/schistosomiasis

24. Zandian A, Forsström B, Häggmark-Månberg A, Schwenk JM, Uhlén M, Nilsson P, et al. Whole-Proteome Peptide Microarrays for Profiling Autoantibody Repertoires within Multiple Sclerosis and Narcolepsy. J Proteome Res [Internet]. 2017 Mar 3 [cited 2020 Feb 26];16(3):1300–14. Available from: http://www.ncbi.nlm.nih.gov/pubmed/28121444

25. El-Manzalawy Y, Dobbs D, Honavar VG. In silico prediction of linear B-cell epitopes on proteins. In: Methods in Molecular Biology. Humana Press Inc.; 2017. p. 255–64.

26. Lo YT, Pai TW, Wu WK, Chang HT. Prediction of conformational epitopes with the use of a knowledge-based energy function and geometrically related neighboring residue characteristics. BMC Bioinformatics [Internet]. 2013 Mar 8 [cited 2021 Apr 17];14(SUPPL4):S3. Available from: /pmc/articles/PMC3599093/

27. Handbook for DTA Reviews | Cochrane Screening and Diagnostic Tests [Internet]. [cited 2020 Mar 3]. Available from: https://methods.cochrane.org/sdt/handbook-dta-reviews

28. Abba K, Deeks JJ, Olliaro P, Naing C-M, Jackson SM, Takwoingi Y, et al. Rapid diagnostic tests for diagnosing uncomplicated P. falciparum malaria in endemic countries. Cochrane database Syst Rev [Internet]. 2011 Jul 6 [cited 2020 Mar 3];(7):CD008122. Available from: http://www.ncbi.nlm.nih.gov/pubmed/21735422

29. Huang HS, Tsai CL, Chang J, Hsu TC, Lin S, Lee CC. Multiplex PCR system for the rapid diagnosis of respiratory virus infection: systematic review and meta-analysis. Vol. 24, Clinical Microbiology and Infection. Elsevier B.V.; 2018. p. 1055–63.

30. Moher D, Liberati A, Tetzlaff J, Altman DG, Altman D, Antes G, et al. Preferred reporting items for systematic reviews and meta-analyses: The PRISMA statement. Vol. 151, Annals of Internal Medicine. American College of Physicians; 2009. p. 264–9.

31. George S, Geldhof P, Albonico M, Ame SM, Bethony JM, Engels D, et al. Molecular speciation of soil-transmitted helminths egg isolates collected during six drug efficacy trials in endemic countries. Trans R Soc Trop Med Hyg. 2016;110(11):657.

32. WHO | What is trachoma [Internet]. [cited 2020 Mar 3]. Available from: https://www.who.int/trachoma/disease/en/

33. Campbell JM, Klugar M, Ding S, Carmody DP, Hakonsen SJ, Jadotte YT, et al. Diagnostic test accuracy: Methods for systematic review and meta-analysis. Int J Evid Based Healthc. 2015 Sep 1;13(3):154–62.

34. Whiting PF, Rutjes AWS, Westwood ME, Mallett S, Deeks JJ, Reitsma JB, et al. QUADAS-2: A Revised Tool for the Quality Assessment of Diagnostic Accuracy Studies [Internet]. 2011 [cited 2020 Mar 3]. Available from: www.annals.org

35. Rutter CM, Gatsonis CA. A hierarchical regression approach to meta-analysis of diagnostic test accuracy evaluations. Stat Med Stat Med. 2001;20:2865–84.

36. Reitsma JB, Glas AS, Rutjes AWS, Scholten RJPM, Bossuyt PM, Zwinderman AH. Bivariate analysis of sensitivity and specificity produces informative summary measures in diagnostic reviews. J Clin Epidemiol. 2005 Oct;58(10):982–90.

37. Macaskill P, Gatsonis C, Deeks J, Harbord R, Takwoingi Y. Cochrane Handbook for Systematic Reviews of Diagnostic Test Accuracy Chapter 10 Analysing and Presenting

For peer review only - http://bmjopen.bmj.com/site/about/guidelines.xhtml

Results [Internet]. [cited 2020 Jun 20]. Available from: http://srdta.cochrane.org/.

38. Leeflang MMG, Kraaijpoel N. Systematic reviews of diagnostic test accuracy in CMI. Vol. 24, Clinical Microbiology and Infection. Elsevier B.V.; 2018. p. 1115–6.

39. Freeman SC, Kerby CR, Patel A, Cooper NJ, Quinn T, Sutton AJ. Development of an interactive web-based tool to conduct and interrogate meta-analysis of diagnostic test accuracy studies: MetaDTA. BMC Med Res Methodol. 2019 Apr 18;19(1).

40. Operating Characteristics of a Rank Correlation Test for Publication Bias - PubMed [Internet]. [cited 2020 Jun 20]. Available from: https://pubmed.ncbi.nlm.nih.gov/7786990/

41. Egger M, Smith GD, Schneider M, Minder C. Bias in meta-analysis detected by a simple , graphical test measures of funnel plot asymmetry. Bmj [Internet]. 1997 Sep 13 [cited 2020 Jun 20];315(7109):629–34. Available from: http://www.ncbi.nlm.nih.gov/pubmed/9310563%0Ahttp://www.pubmedcentral.nih.gov/articlerender.fcgi?artid=PMC2127453

42. Bergamaschi G, Fassi EMA, Romanato A, D'Annessa I, Odinolfi MT, Brambilla D, et al. Computational analysis of dengue virus envelope protein (E) reveals an epitope with flavivirus immunodiagnostic potential in peptide microarrays. Int J Mol Sci [Internet]. 2019 Apr 2 [cited 2020 Nov 23];20(8). Available from: https://pubmed-ncbi-nlm-nih-gov.ukzn.idm.oclc.org/31003530/

43. List C, Qi W, Maag E, Gottstein B, Müller N, Felger I. Serodiagnosis of Echinococcus spp. infection: Explorative selection of diagnostic antigens by peptide microarray. PLoS Negl Trop Dis [Internet]. 2010 Aug 3 [cited 2020 Feb 26];4(8):e771. Available from: http://www.ncbi.nlm.nih.gov/pubmed/20689813

44. Noya O, Patarroyo M, Guzman F, de Noya B. Immunodiagnosis of Parasitic Diseases with Synthetic Peptides. Curr Protein Pept Sci [Internet]. 2003 Aug 1 [cited 2020 Mar 3];4(4):299–308. Available from: http://www.eurekaselect.com/openurl/content.php?genre=article&issn=1389-2037&volume=4&issue=4&spage=299

45. Brandão RMS de S, Martins LMS, Monte SJH do. Synthetic Peptides as an Alternative Tool for the Diagnosis of Cryptococcosis. In: Fungal Pathogenicity [Internet]. InTech; 2016 [cited 2020 Feb 26]. Available from: http://www.intechopen.com/books/fungal-pathogenicity/synthetic-peptides-as-an-alternative-tool-for-the-diagnosis-of-cryptococcosis

46. Grötzinger C. Peptide microarrays for medical applications in autoimmunity, infection, and cancer. In: Methods in Molecular Biology [Internet]. Humana Press Inc.; 2016 [cited 2020 Feb 26]. p. 213–21. Available from: http://www.ncbi.nlm.nih.gov/pubmed/26490478

DECLARATIONS

Ethics approval and consent to participate

Not applicable

Consent for publication

For peer review only

522 Not applicable

523 **Availability of data and material**

524 Data sets used in the current study is available for sharing after reasonable request.

525

526 Word count: 3902 words

For peer review only

For peer review only - http://bmjopen.bmj.com/site/about/guidelines.xhtml

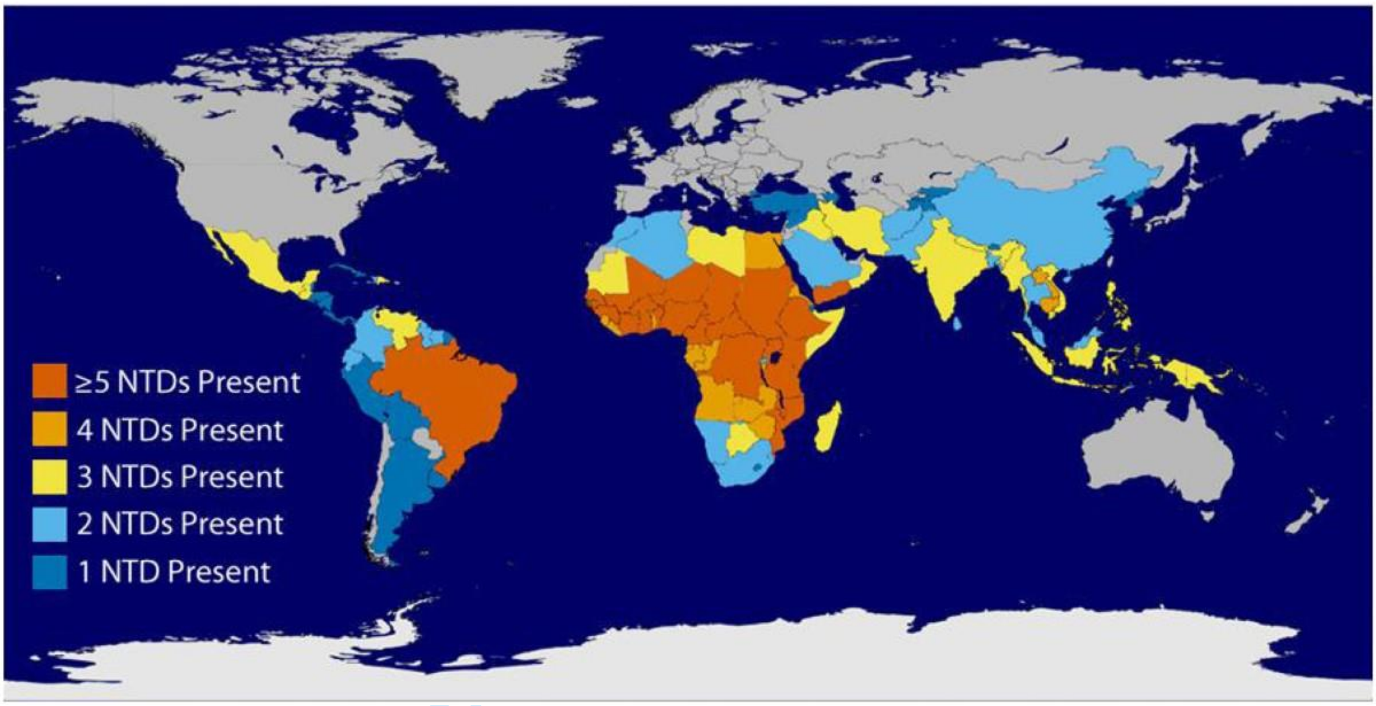

For peer review only

Search	Add to builder	Query	Items found	Time
#16	Add	Search ((((((Microarray Analysis[MeSH Terms]) OR Microarrays) OR Microarray[Title/Abstract]) OR Array[Title/Abstract])) AND ((((Peptides[MeSH Terms]) OR Epitopes[MeSH Terms]) OR Peptide[Title/Abstract]) OR Epitopes[Title/Abstract])) AND ((((Urogenital schistosomiasis) OR Schistosoma haematobium) OR S. haematobium) OR schistosomiasis)	56	04:53:56
#15	Add	Search (((Urogenital schistosomiasis) OR Schistosoma haematobium) OR S. haematobium) OR schistosomiasis	27604	04:53:32
#14	Add	Search schistosomiasis	27038	04:53:06
#13	Add	Search S. haematobium	2547	04:52:51
#12	Add	Search Schistosoma haematobium	3773	04:52:37
#11	Add	Search Urogenital schistosomiasis	2548	04:51:59
#10	Add	Search (((Peptides[MeSH Terms]) OR Epitopes[MeSH Terms]) OR Peptide[Title/Abstract]) OR Epitopes[Title/Abstract]	2902261	04:51:11
#9	Add	Search Epitopes[Title/Abstract]	49899	04:50:47
#8	Add	Search Peptide[Title/Abstract]	406530	04:50:16
#7	Add	Search Epitopes[MeSH Terms]	111708	04:49:55
#6	Add	Search Peptides[MeSH Terms]	2672990	04:49:28
#5	Add	Search (((Microarray Analysis[MeSH Terms]) OR Microarrays) OR Microarray[Title/Abstract]) OR Array[Title/Abstract]	275317	04:48:52
#4	Add	Search Array[Title/Abstract]	146900	04:46:23
#3	Add	Search Microarray[Title/Abstract]	87844	04:45:54
#2	Add	Search Microarrays	30663	04:45:28
#1	Add	Search Microarray Analysis[MeSH Terms]	91509	04:45:05

peer review only

PRISMA-P 2015 Checklist

This checklist has been adapted for use with systematic review protocol submissions to BioMed Central journals from Table 3 in Moher D et al: Preferred reporting items for systematic review and meta-analysis protocols (PRISMA-P) 2015 statement. *Systematic Reviews* 2015 **4**:1

An Editorial from the Editors-in-Chief of *Systematic Reviews* details why this checklist was adapted - Moher D, Stewart L & Shekelle P: Implementing PRISMA-P: recommendations for prospective authors. *Systematic Reviews* 2016 **5**:15

Section/topic	#	Checklist item	Information reported		Line number(s)
			Yes	No	
**ADMINISTRATIVE INFORMATION**					
**Title**					
Identification	1a	Identify the report as a protocol of a systematic review	☒	☐	2 and 3
Update	1b	If the protocol is for an update of a previous systematic review, identify as such	☐	☐	N/A
**Registration**	2	If registered, provide the name of the registry (e.g., PROSPERO) and registration number in the Abstract	☒	☐	46 and 47
**Authors**					
Contact	3a	Provide name, institutional affiliation, and e-mail address of all protocol authors; provide physical mailing address of corresponding author	☒	☐	4-18
Contributions	3b	Describe contributions of protocol authors and identify the guarantor of the review	☒	☐	525-529
**Amendments**	4	If the protocol represents an amendment of a previously completed or published protocol, identify as such and list changes; otherwise, state plan for documenting important protocol amendments	☐	☐	N/A
**Support**					
Sources	5a	Indicate sources of financial or other support for the review	☒	☐	514-524
Sponsor	5b	Provide name for the review funder and/or sponsor	☒	☐	514-524
Role of sponsor/funder	5c	Describe roles of funder(s), sponsor(s), and/or institution(s), if any, in developing the protocol	☒	☐	517-518 and 522-524
**INTRODUCTION**					
**Rationale**	6	Describe the rationale for the review in the context of what is already known	☒	☐	117-120

For peer review only - http://bmjopen.bmj.com/site/about/guidelines.xhtml

Section/topic	#	Checklist item	Information reported Yes	Information reported No	Line number(s)
**Objectives**	7	Provide an explicit statement of the question(s) the review will address with reference to participants, interventions, comparators, and outcomes (PICO)	☒	☐	136-141
**METHODS**					
**Eligibility criteria**	8	Specify the study characteristics (e.g., PICO, study design, setting, time frame) and report characteristics (e.g., years considered, language, publication status) to be used as criteria for eligibility for the review	☒	☐	189-220
**Information sources**	9	Describe all intended information sources (e.g., electronic databases, contact with study authors, trial registers, or other grey literature sources) with planned dates of coverage	☒	☐	160-163 and 181-186
**Search strategy**	10	Present draft of search strategy to be used for at least one electronic database, including planned limits, such that it could be repeated	☒	☐	165-166 and 169-172
STUDY RECORDS					
Data management	11a	Describe the mechanism(s) that will be used to manage records and data throughout the review	☐	☒	
Selection process	11b	State the process that will be used for selecting studies (e.g., two independent reviewers) through each phase of the review (i.e., screening, eligibility, and inclusion in meta-analysis)	☒	☐	225-230
Data collection process	11c	Describe planned method of extracting data from reports (e.g., piloting forms, done independently, in duplicate), any processes for obtaining and confirming data from investigators	☒	☐	231-262
**Data items**	12	List and define all variables for which data will be sought (e.g., PICO items, funding sources), any pre-planned data assumptions and simplifications	☒	☐	272-273
**Outcomes and prioritization**	13	List and define all outcomes for which data will be sought, including prioritization of main and additional outcomes, with rationale	☒	☐	289-306
**Risk of bias in individual studies**	14	Describe anticipated methods for assessing risk of bias of individual studies, including whether this will be done at the outcome or study level, or both; state how this information will be used in data synthesis	☒	☐	263-269
DATA					
**Synthesis**	15a	Describe criteria under which study data will be quantitatively synthesized	☒	☐	282-285
	15b	If data are appropriate for quantitative synthesis, describe planned summary measures, methods of handling data, and methods of combining data from studies, including any planned exploration of consistency (e.g., I^2, Kendall's tau)	☒	☐	285-288 and 301-306

For peer review only

Section/topic	#	Checklist item	Information reported Yes	Information reported No	Line number(s)
	15c	Describe any proposed additional analyses (e.g., sensitivity or subgroup analyses, meta-regression)	☒	☐	312-319
	15d	If quantitative synthesis is not appropriate, describe the type of summary planned	☒	☐	271-281
**Meta-bias(es)**	16	Specify any planned assessment of meta-bias(es) (e.g., publication bias across studies, selective reporting within studies)	☒	☐	307-310
**Confidence in cumulative evidence**	17	Describe how the strength of the body of evidence will be assessed (e.g., GRADE)	☒	☐	264-269

For peer review only
